# GWAS for systemic sclerosis identifies multiple risk loci and highlights fibrotic and vasculopathy pathways

**DOI:** 10.1038/s41467-019-12760-y

**Published:** 2019-10-31

**Authors:** Elena López-Isac, Marialbert Acosta-Herrera, Martin Kerick, Shervin Assassi, Ansuman T. Satpathy, Jeffrey Granja, Maxwell R. Mumbach, Lorenzo Beretta, Carmen P. Simeón, Patricia Carreira, Norberto Ortego-Centeno, Ivan Castellvi, Lara Bossini-Castillo, F. David Carmona, Gisela Orozco, Nicolas Hunzelmann, Jörg H. W. Distler, Andre Franke, Claudio Lunardi, Gianluca Moroncini, Armando Gabrielli, Jeska de Vries-Bouwstra, Cisca Wijmenga, Bobby P. C. Koeleman, Annika Nordin, Leonid Padyukov, Anna-Maria Hoffmann-Vold, Benedicte Lie, European Scleroderma Group†, R. Ríos, J. L. Callejas, J. A. Vargas-Hitos, R. García-Portales, M. T. Camps, A. Fernández-Nebro, M. F. González-Escribano, F. J. García-Hernández, M. J. Castillo, M. A. Aguirre, I. Gómez-Gracia, B. Fernández-Gutiérrez, L. Rodríguez-Rodríguez, P. García de la Peña, E. Vicente, J. L. Andreu, M Fernández de Castro, F. J. López-Longo, L. Martínez, Fonollosa, A. Guillén, G. Espinosa, C. Tolosa, A. Pros, M. Rodríguez-Carballeira, F. J. Narváez, M. Rubio-Rivas, Ortiz-Santamaría, A. B. Madroñero, M. A. González-Gay, B. Díaz, L. Trapiella, A. Sousa, M. V. Egurbide, P. Fanlo-Mateo, L. Sáez-Comet, F. Díaz, Hernández, E. Beltrán, J. A. Román-Ivorra, E. Grau, J. J. Alegre-Sancho, M. Freire, F. J. Blanco-García, N. Oreiro, T. Witte, A. Kreuter, G. Riemekasten, P. Airó, C. Magro, A. E. Voskuyl, M. C. Vonk, R. Hesselstrand, Susanna Proudman, Wendy Stevens, Mandana Nikpour, Australian Scleroderma Interest Group (ASIG), J. Zochling, J. Sahhar, J. Roddy, P. Nash, K. Tymms, M. Rischmueller, S. Lester, Timothy Vyse, Ariane L. Herrick, Jane Worthington, Christopher P. Denton, Yannick Allanore, Matthew A. Brown, Timothy R. D. J. Radstake, Carmen Fonseca, Howard Y. Chang, Maureen D. Mayes, Javier Martin

**Affiliations:** 1 Institute of Parasitology and Biomedicine López-Neyra, IPBLN-CSIC, Granada, Spain; 2 The University of Texas Health Science Center–Houston, Houston, USA; 3 Center for Personal Dynamic Regulomes, Stanford University School of Medicine, Stanford, CA USA; 4 Howard Hughes Medical Institute, Stanford University, Stanford, CA USA; 5 Referral Center for Systemic Autoimmune Diseases, Fondazione IRCCS Ca’ Granda Ospedale Maggiore Policlinico di Milano, Milan, Italy; 6 Department of Internal Medicine, Valle de Hebrón Hospital, Barcelona, Spain; 7 Department of Rheumatology, 12 de Octubre University Hospital, Madrid, Spain; 8 Department of Internal Medicine, San Cecilio Clinic University Hospital, Granada, Spain; 9 Department of Rheumatology, Santa Creu i Sant Pau University Hospital, Barcelona, Spain; 10 Wellcome Trust Sanger Institute, Hinxton, UK; 11 Department of Genetics and Institute of Biotechnology, University of Granada, Granada, Spain; 12 Arthritis Research UK Centre for Genetics and Genomics, Centre for Musculoskeletal Research, Faculty of Biology, Medicine and Health, Manchester Academic Health Science Centre, The University of Manchester, Oxford Road, Manchester, UK; 13 Department of Dermatology, University of Cologne, Cologne, Germany; 14 Department of Internal Medicine 3, Institute for Clinical Immunology, University of Erlangen-Nuremberg, Erlangen, Germany; 15 Institute of Clinical Molecular Biology, Christian-Albrechts-University of Kiel, Kiel, Germany; 16 Department of Medicine, Università degli Studi di Verona, Verona, Italy; 17 Clinica Medica, Department of Clinical and Molecular Science, Università Politecnica delle Marche and Ospedali Riuniti, Ancona, Italy; 18 Department of Rheumatology, Leiden University Medical Center, Leiden, The Netherlands; 19 Department of Genetics, University Medical Center Groningen, University of Groningen, Groningen, Netherlands; 20 University Medical Center Utrecht, Utrecht, The Netherlands; 21 Division of Rheumatology, Department of Medicine, Karolinska University Hospital, Karolinska Institute, Stockholm, Sweden; 22 Department of Rheumatology, Oslo University Hospital, Oslo, Norway; 23 Department of Medical Genetics, and the Department of Immunology, University of Oslo and Oslo University Hospital, Oslo, Norway; 63 Royal Adelaide Hospital and University of Adelaide, Adelaide, SA Australia; 64 St. Vincent’s Hospital, Melbourne, VIC Australia; 65 The University of Melbourne at St. Vincent’s Hospital, Melbourne, VIC Australia; 72 Department of Medical and Molecular Genetics, King’s College London, London, UK; 73 Centre for Musculoskeletal Research, The University of Manchester, Salford Royal NHS Foundation Trust, Manchester Academic Health Science Centre, Manchester, UK; 74 NIHR Manchester Biomedical Research Centre, Manchester, UK; 75 Centre for Rheumatology, Royal Free and University College Medical School, London, United Kingdom; 76 Department of Rheumatology A, Cochin Hospital, INSERM U1016, Paris Descartes University, Paris, France; 77 Institute of Health and Biomedical Innovation, Queensland University of Technology, Translational Research Institute, Princess Alexandra Hospital, Brisbane, QLD Australia; 78 Department of Rheumatology & Clinical Immunology, Laboratory of Translational Immunology, department of Immunology, University Medical Center Utrecht, Utrecht, The Netherlands; 24 Department of Internal Medicine, Virgen de las Nieves Hospital, Granada, Spain; 25 Department of Rheumatology, Virgen de la Victoria Hospital, Málaga, Spain; 26 Department of Internal Medicine, Carlos Haya Hospital, Málaga, Spain; 27 Department of Rheumatology, Carlos Haya Hospital, Málaga, Spain; 28 Department of Immunology, Virgen del Rocío Hospital, Sevilla, Spain; 29 Department of Internal Medicine, Virgen del Rocío Hospital, Sevilla, Spain; 30 Department of Rheumatology, Reina Sofía/IMIBIC Hospital, Córdoba, Spain; 31 Department of Rheumatology, San Carlos Clinic Hospital, Madrid, Spain; 32 Department of Rheumatology, Madrid Norte Sanchinarro Hospital, Madrid, Spain; 33 Department of Rheumatology, La Princesa Hospital, Madrid, Spain; 34 Department of Rheumatology, Puerta de Hierro Hospital-Majadahonda, Madrid, Spain; 35 Department of Rheumatology, Gregorio Marañón University Hospital, Madrid, Spain; 36 Department of Internal Medicine, Clinic Hospital, Barcelona, Spain; 37 Department of Internal Medicine, Parc Tauli Hospital, Sabadell, Spain; 38 Department of Rheumatology, Hospital Del Mar, Barcelona, Spain; 39 Department of Internal Medicine, Hospital Universitari Mútua Terrasa, Barcelona, Spain; 40 Department of Rheumatology, Bellvitge University Hospital, Barcelona, Spain; 41 Department of Internal Medicine, Bellvitge University Hospital, Barcelona, Spain; 42 Department of Rheumatology, Granollers Hospital, Granollers, Spain; 43 Department of Internal Medicine, Hospital General San Jorge, Huesca, Spain; 44 Epidemiology, Genetics and Atherosclerosis Research Group on Systemic Inflammatory Diseases, DIVAL, University of Cantabria, Santander, Spain; 45 Department of Internal Medicine, Hospital Central de Asturias, Oviedo, Spain; 46 Infectious Diseases Unit, Department of Internal Medicine, Hospital Xeral-Complexo Hospitalario Universitario de Vigo, Vigo, Spain; 47 Department of Internal Medicine, Hospital Universitario Cruces, Barakaldo, Spain; 48 Department of Internal Medicine, Hospital Virgen del Camino, Pamplona, Spain; 49 Department of Internal Medicine, Hospital Universitario Miguel Servet, Zaragoza, Spain; 50 Department of Rheumatology, Hospital Universitario de Canarias, Tenerife, Spain; 51 Department of Rheumatology, Hospital General Universitario de Valencia, Valencia, Spain; 52 Department of Rheumatology, Hospital Universitari i Politecnic La Fe, Valencia, Spain; 53 Department of Rheumatology, Hospital Universitari Doctor Peset, Valencia, Spain; 54 Department of Internal Medicine, Thrombosis and Vasculitis Unit, Complexo Hospitalario Universitario de Vigo, Vigo, Spain; 55 Department of Rheumatology, INIBIC-Hospital Universitario A Coruña, La Coruña, Spain; 56 Department of Clinical Immunology, Hannover Medical School, Hannover, Germany; 57 Department of Dermatology, Josefs-Hospital, Ruhr University Bochum, Bochum, Germany; 58 Clinic of Rheumatology, University of Lübeck, Lübeck, Germany; 59 Service of Rheumatology and Clinic Immunology Spedali Civili, Brescia, Italy; 60 Department of Rheumatology, VU University Medical Center, Amsterdam, The Netherlands; 61 Department of Rheumatology, Radboud University Nijmegen Medical Center, Nijmegen, Netherlands; 62 Department of Rheumatology, Lund University, Lund, Sweden; 66 Menzies Research Institute Tasmania, University of Tasmania, Hobart, TAS Australia; 67 Department Rheumatology, Monash Medical Centre, Melbourne, VIC Australia; 68 Rheumatology, Royal Perth Hospital, Perth, WA Australia; 69 Research Unit, Sunshine Coast Rheumatology, Maroochydore, QLD Australia; 70 Canberra Rheumatology, Canberra, ACT Australia; 71 Department Rheumatology, The Queen Elizabeth Hospital, Woodville, SA Australia

**Keywords:** Functional genomics, Genome-wide association studies, Systemic sclerosis, Rheumatic diseases

## Abstract

Systemic sclerosis (SSc) is an autoimmune disease that shows one of the highest mortality rates among rheumatic diseases. We perform a large genome-wide association study (GWAS), and meta-analysis with previous GWASs, in 26,679 individuals and identify 27 independent genome-wide associated signals, including 13 new risk loci. The novel associations nearly double the number of genome-wide hits reported for SSc thus far. We define 95% credible sets of less than 5 likely causal variants in 12 loci. Additionally, we identify specific SSc subtype-associated signals. Functional analysis of high-priority variants shows the potential function of SSc signals, with the identification of 43 robust target genes through HiChIP. Our results point towards molecular pathways potentially involved in vasculopathy and fibrosis, two main hallmarks in SSc, and highlight the spectrum of critical cell types for the disease. This work supports a better understanding of the genetic basis of SSc and provides directions for future functional experiments.

## Abstract (web summary)

Systemic sclerosis (SSc) is a heterogeneous chronic autoimmune disease that affects the connective tissue. Here, López-Isac et al. identify 13 new risk loci for SSc as well as loci specific for limited cutaneous and diffuse SSc and, defining credible sets and performing functional annotation, highlight key pathways and cell types for SSc.

## Introduction

Rheumatic diseases are one of the main causes of physical disability of non-mental origin in the Western world according to the World Health Organization. Rheumatic diseases have a marked impact on the quality of life of patients. Among them, systemic sclerosis (SSc) has one of the highest mortality rates^1^. SSc is a chronic autoimmune disease (AD) that affects the connective tissue, with very heterogeneous clinical manifestations. The pathogenesis of the disease involves extensive fibrosis of the skin and internal organs, vascular damage, and immune imbalance, including autoantibody production^2,3^. Lung involvement—both pulmonary hypertension and/or pulmonary fibrosis—is the leading cause of death^4^.

As most ADs, SSc has a complex genetic component and its etiology is poorly understood. Genome-wide association studies (GWASs) have been successful in the identification of thousands of genetic variants associated with the susceptibility of complex traits. Moreover, GWASs provide invaluable information on disease aetiopathogenesis and contribute to drug discovery and repurposing^5,6^. Several GWASs of SSc have been published, which greatly contributed to the understanding of SSc pathogenesis, and pointed out to relevant pathways for the disease, such as the interferon pathway, the interleukin 12 pathway, and apoptosis^7–12^. Nonetheless, the rate of discovery of previous studies was limited owing to the relatively small sample sizes of the study cohorts.

To continue unraveling the partially known genetic background of SSc, we perform a powerful meta-GWAS in European population that includes ~ 10,000 patients. We also hypothesize that an integrative approach combining all SSc association signals, fine-mapping, and the identification of target genes based on chromatin contacts would provide further insights into the biology of the disease.

## Results

### Twenty-seven signals independently associated with SSc

We performed genome-wide association analyses in 14 independent European cohorts comprising a total of 26,679 individuals (9,095 SSc patients and 17,584 healthy controls). Nine out of the 14 SSc GWAS cohorts were so far unreported, whereas 5 had been previously published^7,8^ (Supplementary Data 1). After correcting for sex and the first five principal components (PCs) (Methods), we did not observe genomic inflation in any of the independent GWAS cohorts, with the exception of the Italian cohort, which remained with residual inflation (Supplementary Data 1 and Supplementary Fig. 1). Overall, the meta-analysis showed a genomic inflation factor (*λ*) of 1.10, with a rescaled *λ*_1000_ of 1.008 for an equivalent study of 1000 cases/1000 controls (Supplementary Data 1).

**Fig. 1:**
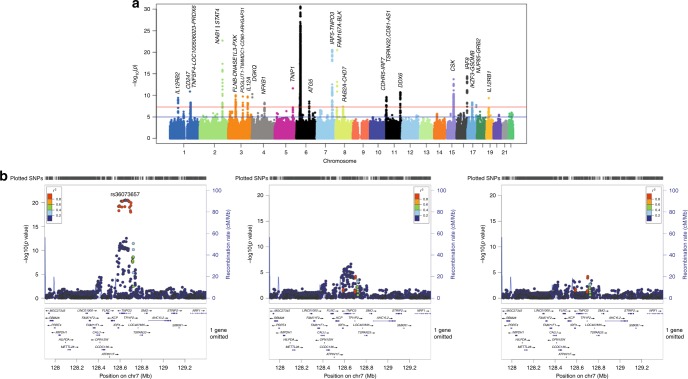
Association signals for systemic sclerosis in a large meta-GWAS. **a** Manhattan plot representing the meta-GWAS results. The −log_10_ of the *p* values are plotted against their physical chromosomal position. The red and blue lines represent the genome-wide level of significance (*p* < 5 × 10^−8^) and *p* value threshold at *p* < 1 × 10^−5^, respectively. The plot has been truncated at *p* < 1 × 10^−30^. The lowest *p* value was observed within the *MHC* region for rs6457617 (association test *p* = 3.25 × 10^−43^). **b** Locuszoom to depict independent association signals in *IRF5-TNPO3*. From left to right, locuszoom of the association signals in *IRF5-TNPO3* for the global meta-analysis; association signals conditioned on the lead SNP (rs36073657), and conditioned on rs36073657 and the secondary signal at the locus (rs12155080)

We undertook an inverse variance-weighted meta-analysis with a high-density genotyped and imputed SNP panel (4.72 million SNPs) to combine all independent GWASs. We considered all the SNPs that were shared by at least two data sets to avoid SNP data loss. This approach yielded 431 significantly associated SNPs (association test *p* value ≤ 5 × 10^−8^) excluding the well-known HLA region. Significant signals involved 23 genomic regions, of which 13 were new genome-wide significant loci for SSc and 10 corresponded to previously reported GWAS signals (Table 1, Fig. 1).

**Table 1:** Twenty-seven signals independently associated with systemic sclerosis in the meta-GWAS

Chr	Locus	Bp	SNP	Index SNP	Ref.	MAF	*N*	*P* value	OR	*Q*	*I*	*P*_cond_	Func refgene
1	*IL12RB2*	67814440	rs3790566	Yes	T	0.24	13	3.84E-10	1.16	0.80	0	-	Intronic
1	*CD247*	167420425	rs2056626	Yes	G	0.39	6	1.31E-11	0.81	0.57	0	-	Intronic
**1**	***TNFSF4-*** ***LOC100506023*** ***-PRDX6***	**173238736**	**rs2022449**	**No**	**T**	**0.23**	**12**	**6.28E-08**	**1.15**	**0.90**	**0**	**6.63E-08**	**Regulatory region**
**1**	***TNFSF4-*** ***LOC100506023*** ***-PRDX6***	**173332629**	**rs1857066**	**Yes**	**A**	**0.25**	**13**	**5.02E-09**	**0.87**	**0.84**	**0**	-	**ncRNA intronic**
**2**	NAB1*	**191534372**	**rs16832798**	**Yes**	**C**	**0.14**	**14**	**5.20E-09**	**1.18**	**0.41**	**3.79**	3.84E-07	**Intronic**
2	*STAT4*	191902758	rs3821236	Yes	A	0.20	12	1.94E-23	1.31	0.03	48.21	-	Intronic
2	*STAT4*	191959489	rs4853458	No	A	0.23	9	4.86E-18	1.35	0.42	1.79	5.58E-08	Intronic
3	*FLNB* *-DNASE1L3-PXK*	58131515	rs7355798	No	T	0.24	13	1.24E-08	1.14	0.14	30.5	7.42E-07	Intronic
3	*FLNB-DNASE1L3-* *PXK*	58375286	rs4076852	Yes	G	0.26	13	1.04E-10	1.16	0.71	0	-	Intronic
**3**	***POGLUT1-TIMMDC1-CD80-*** ***ARHGAP31***	**119116150**	**rs9884090**	**Yes**	**A**	**0.16**	**13**	**1.89E-10**	**0.83**	**0.92**	**0**	-	**Intronic**
3	*IL12A*	159733527	rs589446	Yes	T	0.35	11	1.95E-10	0.86	0.85	0	-	ncRNA intronic
**4**	***DGKQ***	**965779**	**rs11724804**	**Yes**	**A**	**0.44**	**12**	**5.31E-11**	**1.17**	**0.24**	**21.04**	-	**Intronic**
**4**	***NFKB1***	**103449041**	**rs230534**	**Yes**	**T**	**0.34**	**10**	**5.38E-09**	**1.15**	**0.92**	**0**	-	**Intronic**
5	*TNIP1*	150455732	rs3792783	Yes	G	0.16	14	2.42E-12	1.20	0.03	47.41	-	Intronic
6	*ATG5*	106734040	rs633724	Yes	T	0.35	14	2.84E-09	1.13	0.31	13.41	-	Intronic
7	*IRF5-* *TNPO3*	128651522	rs36073657	Yes	T	0.10	12	3.10E-21	1.40	0.21	23.35	-	Intronic
7	*IRF5-* *TNPO3*	128658739	rs12155080	No	G	0.37	13	2.87E-13	0.85	0.69	0	2.22E-07	Intronic
**8**	***FAM167A-BLK***	**11343973**	**rs2736340**	**Yes**	**T**	**0.24**	**14**	**3.33E-21**	**1.24**	**0.17**	**26.76**	-	**Intergenic**
**8**	***RAB2A-CHD7***	**61564964**	**rs685985**	**Yes**	**T**	**0.47**	**11**	**3.82E-08**	**0.87**	**0.15**	**30.84**	-	**Intergenic**
**11**	***CDHR5*** ***-IRF7***	**618172**	**rs6598008**	**Yes**	**A**	**0.44**	**4**	**1.97E-08**	**0.80**	**0.16**	**42.27**	-	**Intronic**
**11**	***TSPAN32,CD81-AS1***	**2348619**	**rs2651804**	**Yes**	**T**	**0.17**	**12**	**2.54E-10**	**0.82**	**0.67**	**0**	-	**Intergenic**
**11**	***DDX6***	**118639353**	**rs11217020**	**Yes**	**A**	**0.20**	**14**	**2.08E-11**	**0.84**	**0.80**	**0**	-	**Intronic**
15	*CSK*	75077367	rs1378942	Yes	C	0.39	13	1.84E-14	1.18	0.90	0	-	Intronic
**16**	***IRF8***	**85971922**	**rs11117420**	**Yes**	**C**	**0.19**	**12**	**3.82E-15**	**0.81**	**0.47**	**0**	-	**Intergenic**
**17**	***IKZF3*** ***-GSDMB***	**38063381**	**rs883770**	**Yes**	**T**	**0.50**	**14**	**4.79E-09**	**1.13**	**0.75**	**0**	-	**Intronic**
**17**	***NUP85*** ***-GRB2***	**73224639**	**rs1005714**	**Yes**	**G**	**0.20**	**13**	**1.87E-08**	**0.85**	**0.68**	**0**	-	**Intronic**
19	*IL12RB1*	18193191	rs2305743	Yes	A	0.20	12	4.64E-10	0.83	0.28	16.88	-	Intronic

The new genome-wide significant loci for systemic sclerosis are highlighted in bold. *NAB1*-rs16832798 *p* value conditioned on conditioned on *STAT4*-rs3821236 and *STAT4*-rs4853458. For those intronic or regulatory SNPs that are located in a high gene density region, the gene they lie in was underlined

*Bp* base pair, *Chr* chromosome, *MAF* minor allele frequency in the 1000 Genome Project European Population, *N* number of cohorts, *OR* odds ratio, P*cond* *p* value conditioned on index SNP, *Ref.* reference allele, *SNP* single-nucleotide polymorphism

The presence of independent signals in the genomic regions that showed significant associations was investigated by stepwise conditional analysis using summary statistics from the meta-analysis (Methods). Four genomic regions—*TNFSF4 (1q25.1)*, *STAT4 (2q32.2-q32.3)*, *DNASE1L3 (3p14.3)*, and *IRF5-TNPO3 (7q32.1)*—showed additional significant signals after conditioning on the lead SNP of each locus (conditional association test *p* value (*P*cond) <  5  ×  10^−6^) (Table 1, Fig. 1, Supplementary Fig. 2). Hence, a total of 27 independent signals associated with SSc were identified.

The two independent signals identified in the *DNASE1L3* genomic region (3p14.3) (rs4076852, rs7355798) were intronic variants at *PXK* and *FLNB*, respectively. *PXK*-rs4076852 is in high linkage disequilibrium (LD) with *PXK*-rs2176082 (*r*^2^ = 0.92), which was reported to be associated with SSc in Martin et al.^13^ However, two Immunochip studies conducted by Mayes et al.^9^ and Zochling et al.^12^ showed that the primary association in this genomic region was with the nonsynonymous SNP *DNASE1L3*-rs35677470 (R206C), not present in our SNP panel. Mayes et al.^9^ showed that the *PXK*-rs2176082 association was dependent on the rs35677470 (R206C). Therefore, although we could not analyze the dependence in our GWAS data, we presumed that *PXK*-rs4076852 signal was also dependent on *DNASE1L3*-rs35677470 on the basis of previous evidence. Regarding the intronic signal in *FLNB* (rs7355798), we could not estimate whether it was dependent on *DNASE1L3*-rs35677470 or not. However, given its role in vascular injury repair^14^, *FLNB* may be an interesting SSc locus and should be the object of future research. In the case of the *STAT4* genomic region (2q32.2-q32.3), we observed three independent signals, of which the third was an intronic variant at *NAB1* (rs16832798). This finding—added to further functional evidence provided below—revealed *NAB1* as a new SSc risk locus. We also observed that the genome-wide signal in *GSDMB* (17q21.1; rs883770) was independent (*P*cond = 1.27  ×  10^−7^) from the recently reported signal at *GSDMA* (rs3894194), which is located in the same genomic region^10^.

### Fine-mapping of SSc-associated loci in a Bayesian framework

The identification of the causal SNPs driving the association signals remains an open question after completion of a GWAS. To address this question, Bayesian fine-mapping was performed to define 95% credible sets (the smallest set of variants that summed together at least a 95% probability of including the likely causal variant) in each of the independently associated loci (the two independent signals in *IRF5-TNPO3* were excluded as fine-mapping was not feasible). To improve SNP prioritization accuracy, the probabilistic method integrated association strength with functional annotation data (Methods). Eighteen (72%) and 12 (48%) out of the 25 loci were fine-mapped to ≤10 and to <5 plausible causal variants, respectively (Table 2, Supplementary Data 2). In six loci, the 95% credible set comprised a single variant (*ARHGAP31*, *BLK*, *CD247*, *TNIP1*, *CSK*, *STAT4*-a), and for four others the credible set contained two SNPs (*DGKQ*, *NUP85*-*GRB2*, *STAT4*-b, *IL12RB1*). Moreover, in 64% of the credible sets, the index SNP showed the maximum posterior probability (PP_max_) of being causal. The SNPs with PP_max_ were intergenic, intronic, or noncoding RNA intronic (ncRNA intronic) variants, although the remaining credible set SNPs involved additional SNP categories, namely: UTR3′, downstream, exonic synonymous, and exonic nonsynonymous (Table 2).

**Table 2:** Posterior probabilities of systemic sclerosis fine-mapped loci

Chr	Credible set locus	SNPs Cred. Set	Index SNP	PP Index	SNP PP Max	PP Max	Func.refgene SNP PP Max	Func.refgene in the 95% credible set
1	*IL12RB2*	6	rs3790566	0.195	rs3790567	0.321	Intronic	Intronic
1	*CD247*	1	rs2056626	0.999	rs2056626	0.999	Intronic	-
1	*TNFSF4-* *LOC100506023* *-PRDX6*	6	rs2022449	0.659	rs2022449	0.659	ncRNA Intronic	ncRNA_intronic
1	*TNFSF4-* *LOC100506023* *-PRDX6*	43	rs1857066	0.046	rs11576547	0.265	ncRNA intronic	ncRNA_intronic
2	*NAB1*	11	rs16832798	0.191	rs716254	0.242	Intronic	Intronic; intergenic; downstream
2	*STAT4-a* ^*1*^	1	rs3821236	1.000	rs3821236	1.000	Intronic	-
2	*STAT4-b* ^*2*^	2	rs4853458	0.905	rs4853458	0.905	Intronic	Intronic
3	*FLNB* *-DNASE1L3-PXK*	6	rs7355798	0.365	rs7355798	0.365	Intronic	Intronic
3	*FLNB-DNASE1L3-* *PXK*	27	rs4076852	0.123	rs7653734	0.292	Intronic	Intronic; intergenic
3	*POGLUT1-TIMMDC1-CD80-* *ARHGAP31*	1	rs9884090	0.956	rs9884090	0.956	Intronic	-
3	*IL12A*	23	rs589446	0.385	rs589446	0.385	ncRNA intronic	ncRNA_intronic
4	*DGKQ*	2	rs11724804	0.793	rs11724804	0.793	Intronic	Intronic
4	*NFKB1*	6	rs230534	0.200	rs230517	0.329	Intronic	Intronic
5	*TNIP1*	1	rs3792783	0.999	rs3792783	0.999	Intronic	-
6	*ATG5*	3	rs633724	0.588	rs633724	0.588	Intronic	Intronic
8	*FAM167A-BLK*	1	rs2736340	1.000	rs2736340	1.000	Intergenic	-
8	*RAB2A-CHD7*	80	rs685985	0.003	rs6987084	0.139	Intronic	Intronic; UTR3; intergenic
11	*CDHR5* *-IRF7*	4	rs6598008	0.760	rs6598008	0.760	Intronic	Intronic; exonic synonymous SNV; UTR3; exonic nonsynonymous
11	*TSPAN32,CD81-AS1*	20	rs2651804	0.184	rs2651804	0.184	Intergenic	Intergenic
11	*DDX6*	7	rs11217020	0.021	rs10892286	0.775	Intronic	Intronic; intergenic
15	*CSK*	1	rs1378942	0.993	rs1378942	0.993	Intronic	-
16	*IRF8*	6	rs11117420	0.202	rs11117422	0.54	Intergenic	Intergenic
17	*IKZF3* *-GSDMB*	17	rs883770	0.032	rs9303277	0.157	Intronic	Intergenic; intronic; exonic synonymous SNV; exonic nonsynonymous
17	*NUP85* *-GRB2*	2	rs1005714	0.940	rs1005714	0.940	Intronic	Intronic
19	*IL12RB1*	2	rs2305743	0.944	rs2305743	0.944	Intronic	Intronic

*Chr* chromosome, *PP* posterior probability, *SNP* single-nucleotide polymorphism

^1^Name of the credible set that comprised the index SNP from *STAT4* genomic region (2q32.2-q32.3)

^2^Name of the credible set that comprised the secondary association signal in *STAT4* genomic region (2q32.2-q32.3)

### Functional annotation of SNPs from credible sets

Since most of the likely causal variants were linked to regulatory functions rather than affecting the function of proteins encoded by surrounding genes, we further explored their regulatory effects. For this purpose, we performed functional annotation of SNPs from credible sets through eQTL analysis (Methods). In addition, we explored overlap with histone marks of active promoters and active enhancers (H3K9ac, H3K4me1, and H3K27ac) of cell types relevant to the disease using data from the Roadmap Epigenomics Project^15^ (Supplementary Table 1) (Methods).

Supplementary Figure 3 summarizes the results of the functional characterization of credible set SNPs. When the 95% credible set was not well resolved (credible sets that contained > 15 likely causal variants), we selected the SNP with PP_max_ and the index SNP. In the case of *IRF5-TNPO3*, where the credible set was not feasible, we selected the two independent signals identified at this locus. We obtained a final reduced list of credible set SNPs containing a total of 81 variants. As it can be observed in Supplementary Fig. 3, the vast majority of the likely causal variants overlapped with promoter and enhancer histone marks in the cell types interrogated. These observations suggest that most of the genetic variations involved in the susceptibility to SSc modulate transcriptional regulatory mechanisms. In this regard, we found that 61 out of the 81 interrogated variants (75.31% of the 81 listed credible set SNPs) represent eQTLs, thus altering gene expression in different tissues and cell types (Supplementary Data 3). In fact, the credible set SNPs were significantly enriched for eQTLs in blood and non-blood tissues (odds ratio (OR) = 3.05, Fisher’s exact test *P* = 5.65 × 10^−6^; OR = 1.61, Fisher’s exact test *P* = 4.48 × 10^−2^, respectively) (Supplementary Table 2). Many SNPs were shown to impact the expression of the closest gene (a priori candidate gene) (Supplementary Data 3). In addition, we also found genetic variants affecting the expression of a priori candidate genes and other genes. However, some SNPs only showed eQTL signals for genes other than the closest one. As an example, the SNP rs9884090—which is an intronic variant at *ARHGAP31*—was found to alter the expression of *POGLUT1* and *TIMMDC1* in several tissues (Supplementary Data 3). These results highlight that assigning association signals to the nearby gene is not always the most appropriate strategy and the functional role of certain SSc signals may expand to different target genes.

Five 95% credible sets comprised exonic nonsynonymous variants or contained SNPs in high-to-moderate LD with exonic nonsynonymous variants (*r*^2^ ≥ 0.8, *r*^2^ ≥ 0.6, respectively) (*ARHGAP31*, *IRF7*, *GSDMB*, *NUP85-GRB2,* and *IL12RB1*) (Supplementary Fig. 3, Supplementary Data 4). However, based on SIFT and PolyPhen, none of these exonic variants showed a clear consensus to be deleterious^16,17^ (Supplementary Data 4).

Finally, we assessed pleiotropic effect of our signals by determining whether the likely causal SNPs were also risk factors for other diseases. The results showed extensive overlap especially with other two ADs: systemic lupus erythematosus and primary biliary cholangitis (Supplementary Data 5). These findings were consistent with previous reports that identified shared risk loci for SSc and other immune-mediated diseases^11,13^.

### H3K27ac HiChIP in T cells expands and refines target genes

As stated above, assigning disease-associated variants to the closest gene is not always an appropriate strategy to determine the potential mechanistic effect of association signals. With the aim of identifying the putative drivers of SSc association hits on the basis of functional evidence, we performed an analysis of experimentally derived high-resolution maps of enhancer-promoter interactions generated by H3K27ac HiChIP experiments in human CD4 + T cells^18^ (Methods).

**Fig. 2:**
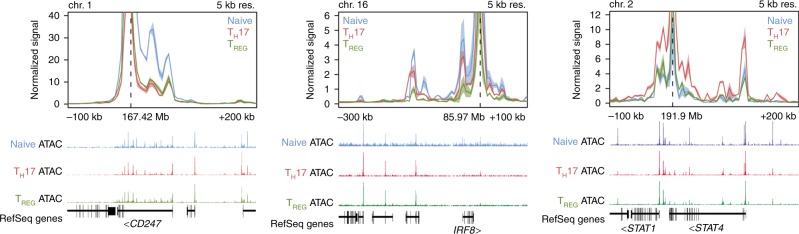
H3K27ac HiChIP signals at systemic sclerosis loci in human CD4^+^ T cells. The SNPs with maximum Posterior Probabilities in each locus were set as anchor points to assess promoter-enhancer chromatin interactions. Representation of overlap with ATAC-seq peaks is included. Chr, chromosome; Kb, kilo base; Mb, mega base; Res, resolution

HiChIP interactions were detected in 18 out of the 27 (66.67%) independently associated loci, using the SNPs with PP_max_ as anchor points (Table 3). Several intronic variants were linked to the target gene promoter in which they are mapped. This was the case of *CD247*-rs2056626, which showed a strong H3K27ac HiChIP signal to the *CD247* promoter (Fig. 2). Other relevant examples of this type of interactions were found in *IL12RB2* and *NFKB1*. The intronic variants in *STAT4*, rs3821236 and rs4853458, showed strong normalized HiChIP signal to *STAT4* and *STAT1* promoters (Fig. 2). We also observed HiChIP contacts that linked intergenic SNPs to the closest genes. For example, rs11117422, located ~40 kb downstream of *IRF8* transcriptional start site, showed interactions with the promoter region of *IRF8* (Fig. 2). In addition, several other enhancer-promoter interactions linked intronic and intergenic SNPs to distant genes. In total, H3K27ac HiChIP signals nominated 155 target genes from 18 SSc likely causal variants (~8 genes per SNP on average) (Table 3).

**Table 3:** H3K27ac HiChIP target genes and nominated target genes for the 27 systemic sclerosis association signals

Ch	Locus	SNP PP Max	HiChIP target genes with SNP PP Max	Nominated genes by H3K27ac HiChIP + eQTL validation	Nominated genes by CHi-C + eQTL validation	Prioritized genes by DEPICT/other criteria
1	*IL12RB2*	rs3790567	***IL12RB2*** *, IL23R*	*IL12RB2*	*IL12RB2, SERBP1*	*IL12RB2*
1	*CD247*	rs2056626	***CD247*** *, POU2F1, TADA1, GPA33, MAEL, DUSP27, CREG1, RCSD1, MPZL1, DCAF6, MPC2*	*CD247*	*CD247*	*CD247*
1	*TNFSF4-* *LOC100506023* *-PRDX6*	rs2022449				*TNFSF4*
1	*TNFSF4-* *LOC100506023* *-PRDX6*	rs11576547				*TNFSF4* ^*a*^
2	*NAB1*	rs716254	***NAB1*** *, GLS, TMEM194B, MFSD6*	*NAB1*		*NAB1, TMEM194B*
2	*STAT4*	rs3821236	*STAT4, STAT1, GLS, MYO1B, NABP1, SDPR*	STAT4, STAT1**		*STAT4*
2	*STAT4*	rs4853458	*STAT4, STAT1, GLS, MYO1B, NABP1, SDPR*	STAT4, STAT1**		
3	*FLNB* *-DNASE1L3-PXK*	rs7355798			*PXK, FLNB, RPP14*	*FLNB-AS1*
3	*FLNB-DNASE1L3-* *PXK*	rs7653734			*PXK, RPP14*	*PXK, DNASE1L3* ^*b*^
3	*POGLUT1-TIMMDC1-CD80-* *ARHGAP31*	rs9884090			*POGLUT1, TIMMDC1*	*CD80, ARHGAP31, POGLUT1*
3	*IL12A*	rs589446	***IL12A***	*IL12A*	*IL12A*	
4	*DGKQ*	rs11724804			*DGKQ, GAK, TMEM175*	
4	*NFKB1*	rs230517	***NFKB1, MANBA*** *, UBE2D3, CISD2, SLC9B1, SLC39A8*	*NFKB1, MANBA*	*NFKB1, MANBA, BDH2*	*NFKB1*
5	*TNIP1*	rs3792783	***TNIP1****, GPX3, CD71, RPS14, RMB22, DCTN4, IRGM, SMIM3*,***ANXA6****, GM2A, SLC36A3*	*TNIP1, ANXA6*	*TNIP1, ANXA6*	*TNIP1*
6	*ATG5*	rs633724				*ATG5* ^*b*^
7	*IRF5-* TNPO3*	rs36073657	*TNPO3*, ***IRF5***	*IRF5*	*IRF5, FAM71F2*	
7	*IRF5-* TNPO3*	rs12155080	***TNPO3***, ***IRF5***	*TNPO3, IRF5*	*TNPO3*	*IRF5*
8	*FAM167A,BLK*	rs2736340			*BLK, RP11-481A20.11, FDFT1, NEIL2*	*BLK, C8orf14*
8	*RAB2A-CHD7*	rs6987084	*CHD7*, ***RAB2A****, CLVS1*	*RAB2A*		
11	*CDHR5* *-IRF7*	rs6598008	***IRF7****, RIC8A, BET1L, PSMD13, SIRT3, NLRP6, PTDSS2*, ***RNH1***, ***HRAS****, RASSF7*, ***PHRF1****, DEAF1****, DRD4****, TALDO1, PDDC1, CEND1*, ***EPS8L2***	*IRF7, RNH1, HRAS, PHRF1, DRD4, EPS8L2*	*DRD4, C11orf35, IRF7, PHRF1, RNH1*	*IRF7*
11	*TSPAN32,CD81-AS1*	rs2651804			*CD81, ASCL2*	*TSPAN32*
11	*DDX6*	rs10892286	***DDX6****, KMT2A*, ***TREH****, CXCR5, BCL9L, UPK2, ATP5L, TRAPPC4, FOXR1, VPS11*	*DDX6, TREH*	*PHLDB1, TREH*	
15	*CSK*	rs1378942	***CSK****, PML, STOML1, ARID3B, SEMA7A, CLK3, EDC3*, ***CYP1A1***, ***LMAN1L***, ***CPLX3***, ***ULK3***, ***SCAMP2***, ***MPI****, COX5A*, ***RPP25***, ***PPCDC****, GOLGA6C, MAN2C1, NEIL1, SIN3A, SNUPN, SNX33*	*CSK, CYP1A1, LMAN1L, CPLX3, ULK3, SCAMP2, MPI, RPP25, PPCDC*	*CPLX3, CSK, CYP1A1, FAM219B, LMAN1L, MPI, PPCDC, RPP25, SCAMP2, SCAMP5, ULK3*	*CSK, SCAMP2, ULK3*
16	*IRF8*	rs11117422	***IRF8*** *, COX4I1, EMC8, FOXF1*	IRF8**		*IRF8*
17	*IKZF3-* *GSDMB*	rs9303277	***GSDMB***, ***IKZF3***, ***ZPBP2****, GRB7, MED1, CDK12, PPP1R1B,****PNMT***, ***PGAP3****, ERBB2*, ***ORMDL3****, NEUROD2, STARD3, TCAP, MIEN1, RPL19, CACNB1, FBXL20, LRRC3C*, ***GSDMA***, ***PSMD3****, THRA, NR1D1, MSL1, RARA, GJD3, TNS4, IGFBP4, CCR7, SMARCE1, KRTAP17, KRT33A*	*GSDMB, IKZF3, ZPBP2, PNMT, PGAP3, ORMDL3, GSDMA, PSMD3*	*IKZF3, ZPBP2, GSDMB, ORMDL3*	*GSDMB, IKZF3, ORMDL3*
17	*NUP85-* *GRB2*	rs1005714	***NUP85***, ***MRPS7****, ATP5H, ICT1*	*NUP85, MRPS7*	*ARMC7, GGA3, SLC16A5, MRPS7, NT5C, NUP85*	*ARMC7, GGA3, NT5C*
19	*IL12RB1*	rs2305743	***IL12RB1****, MAST3, ARRDC2, ISYNA1, ELL, SSBP4, LSM4, JUND, RAB3A, PDE4C*, ***KIAA1683****, MPV17L2, IFI30, PIK3R2, B3GNT3, FCHO1, INSL3, JAK3, RPL18A, SLC5A*	*IL12RB1, KIAA1683*	*KIAA1683*	*IL12RB1, MAST3*

In column ‘HiChIP Target Genes withSNP PP Max’, genes with HiChIP and eQTL signals are highlighted in bold

Ch, chromosome; PP, posterior probability from the statistical fine-mapping; SNP, single-nucleotide polymorphism

*Credible set not feasible

**NO eQTL signals found but strong HiChIP signal

^a^Gene nominated by proximity

^b^Genes nominated according to the results from the first Immunochip in systemic sclerosis (9)

Subsequently, we further validated the functional relevance of H3K27ac HiChIP results by investigating whether the explored SNPs were eQTLs for the nominated target genes. Forty enhancer-target gene relationships showed overlap with SSc eQTL genes (eGenes) (OR = 10.1, Fisher’s exact test *P* = 2.92 × 10^−19^, Supplementary Table 3). Although no eQTL to *IRF8*, *STAT4,* and *STAT1* signals were found, the enrichment of the HiChIP signal observed at these loci (*q* value < 1e-60, Methods) over a global background of distance-matched interactions, and the crucial role of these genes in the immune response, provided evidence to prioritize them as candidate genes. Remarkably, the third independent association signal observed in the *STAT4* genomic region (2q32.2-q32.3)—mapped in a *NAB1* intron—was linked to *NAB1* promoter by our H3K27ac HiChIP analysis. The interaction was validated by an eQTL signal (Supplementary Data 3). These results supported *NAB1* as a new SSc risk locus.

In total, we provided strong evidence to nominate 43 genes as robust SSc target genes in CD4 + T cells (Table 3). Interestingly, some of them pinpointed to new mechanistic insights relevant for the diseases (see Discussion).

### Chromatin interaction analyses in other relevant cell types

It is noteworthy that the epigenomic profiles are cell type specific^19,20^. Considering that the HiChIP analyses were performed in CD4^+^ T cells, and that the pathogenesis of SSc is not only mediated by T cells, we also explored chromatin interaction maps derived from promoter capture Hi-C experiments in additional immune cell types^21,22^ (Methods). These analyses identified promoter interactions not observed in CD4^+^ T cells, which targeted new genes (Table 3, Supplementary Data 6). For example, we observed that the *FLNB*-intronic variant rs7355798 interacted with *FLNB* and *PXK* promoters in B cells and macrophages. Moreover, some of the interactions observed with H3K27ac HiChIP in CD4^+^ T cells were also found in other immune cell types.

The functional relevance of the observed chromatin interactions by promoter capture Hi-C analyses was also validated by eQTLs analysis. In total, these analyses nominated 25 additional target genes for SSc (Table 3).

### Candidate genes prioritized by DEPICT

In addition, we also conducted gene prioritization by means of DEPICT (Data-driven Expression-Prioritized Integration for Complex Traits) (http://www.broadinstitute.org/mpg/depict/index.html), which pinpoints the most likely candidate gene/s in associated loci based on predicted gene functions^23^. Significant gene prioritization *p* values were found in 19 of the 27 queried loci (Table 3, Supplementary Data 7). Most of the prioritized genes were previously nominated by the chromatin interaction analyses. In addition, this method nominated *TNFSF4*, *TMEM194B*, *FLNB*-*AS1, CD80*, *ARHGAP31*, *C8orf14*, *TSPAN32,* and *MAST3*.

### Tissue-specific enrichment of SSc loci in epigenetic marks

The majority of credible set SNPs overlapped with epigenetic marks related to active regions (Supplementary Fig. 3). To quantify the extent of this overlap, we investigated whether SSc associations were non-randomly distributed in histone marks of active promoters (H3K9ac, H3K4me2, H3K4me3, H3K4ac), active enhancers (H3K27ac, H3K4me1, H2BK20ac), and active (or at least accessible) genes (H3K79me1, H2BK15ac) across the 127 reference epigenomes available from the Roadmap Epigenomics Consortium and the Encyclopedia of DNA Elements (ENCODE) projects^15,24^. We used a nonparametric approach (GARFIELD^25,26^) to compute ORs and estimate the significance of functional enrichment at various GWAS *p* value cutoffs (5 × 10^−6^, 5 × 10^−7^, and 5 × 10^−8^) (Methods).

**Fig. 3:**
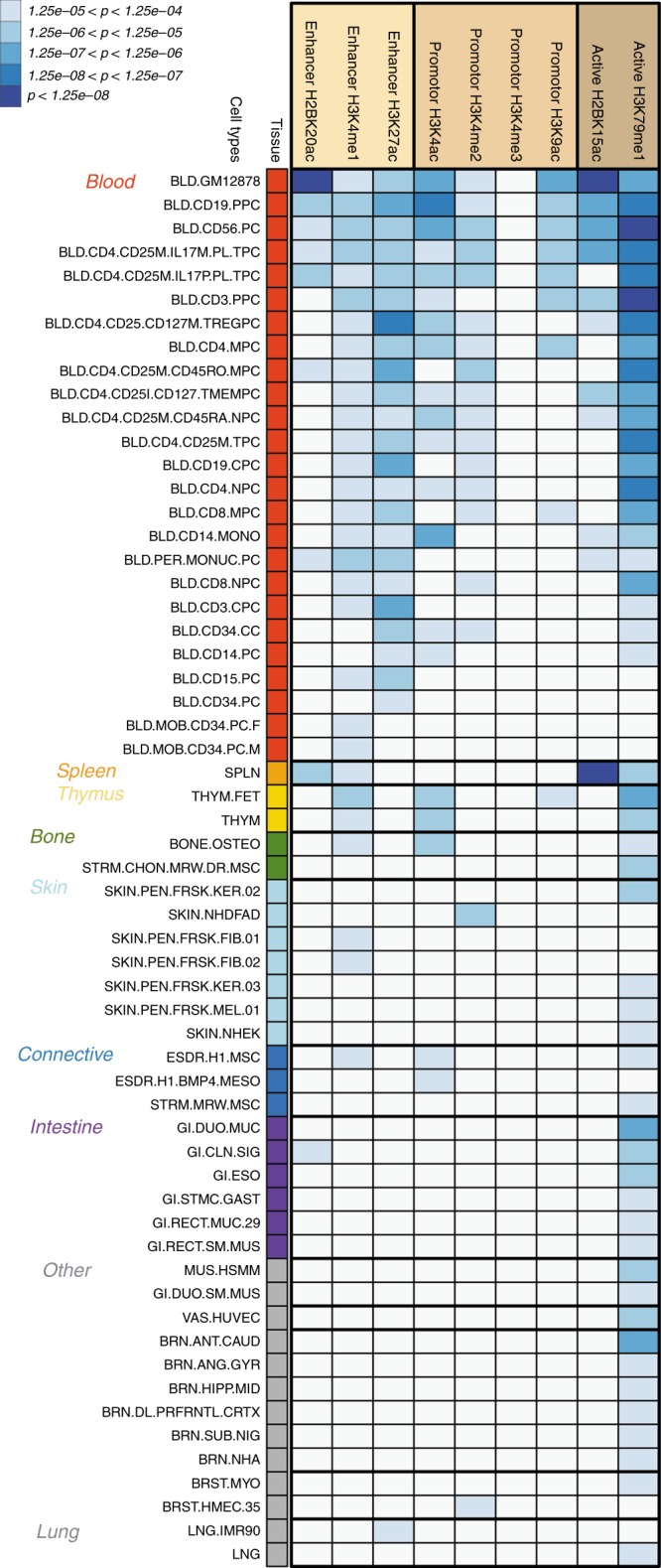
Tissue-specific enrichment for systemic sclerosis associations in epigenetic marks. The heatmap displays the significant enrichment (*p* value < 1.25 × 10^−4^) in 59 out of the 127 cell and tissue types in Roadmap Epigenomics Consortium and the Encyclopedia of DNA Elements (ENCODE) projects. The enrichment *p* values are plotted with different colors according to the strength of the significance. Since the enrichments were computed at various GWAS *p* value cutoffs (5 × 10^−6^, 5 × 10^−7^, 5 × 10^−8^), the most significant *p* value was selected if a cell type/epigenetic mark combination showed more than one significant enrichment across the different cutoffs. Supplementary Data 13 provides the correspondence between cell codes and cell types

Our results showed 363 significant enrichments (*p* value < 1.25 × 10^−4^) in 59 out of the 127 cell and tissue types analyzed (Fig. 3, Supplementary Data 8). Most of the significant enrichments were found in immune cells. SSc-associated variants displayed the most significant enrichments in H2BK15ac and H2BK20ac marks in the GM12878 lymphoblastoid cell line (OR = 37.33, enrichment *p* value (*P*_enr_) = 4.84 × 10^−14^; OR = 12.79, *P*_enr_ = 2.40 × 10^−10^, respectively), followed by H3K79me1 in primary Natural Killer (NK) cells (BLD.CD56.PC) (OR = 12.23, *P*_enr_ = 5.73 × 10^−09^) and primary T cells (BLD.CD3.PPC) (OR = 12.58, *P*_enr_ = 9.78 × 10^−09^). The spleen also showed a strong functional enrichment in H2BK15ac (OR = 14.69, *P*_enr_ = 9.81 × 10^−09^). There were significant enrichments of associations with SSc within H3K27ac, H3K4me1, H3K4me2, and H3K9ac marks—among others—of several CD4^+^ T cells (T helper, T regulatory, etc), CD8^+^ T cells, primary B cells, monocytes, primary neutrophils, and thymus (Fig. 3, Supplementary Data 8). Moreover, the SSc association signals showed different epigenetic enrichment patterns in non-immune cell/tissue types, such as lung, fibroblasts, chondrocytes, keratinocytes, osteoblasts, intestinal mucosa, and esophagus, among others (Fig. 3, Supplementary Data 8).

Interestingly, our large panel of reference epigenomes allowed us to identify cell type specific patterns of enrichment. This specificity was especially relevant for some tissue/cell types that showed enrichment for a single histone mark. For example, dermal fibroblast primary cells (SKIN.NHDFAD) only showed significant enrichment for H3K4me2 (OR = 7.91, *P*_enr_ = 6.54 × 10^−6^).

### Specific patterns of associations for the main SSc subtypes

We performed GWAS stratified analyses considering the main SSc clinical subtypes (limited cutaneous SSc (lcSSc) or diffuse cutaneous SSc (dcSSc)) and autoantibody status according to the presence of anticentromere (ACA), antitopoisomerase (ATA), and anti-RNA polymerase III (ARA) autoantibodies (Supplementary Table 4) (Methods).

A total of 18 and 5 non-HLA significant signals were identified for lcSSc and dcSSc, respectively, representing 15 new genome-wide significant signals for lcSSc and 3 for dcSSc (Supplementary Data 9). Among the associations, there were loci that yielded stronger associations and larger effect size in the subtype analyses than in the global meta-analysis; all despite the reduction of the sample and, consequently, of statistical power. To assess whether the more powerful genetic signals were randomly observed, we performed 10,000 permutation analyses (Methods) and computed empirical *p* values (p*) taking into account the proportion of permuted genetic signals that were at least as extreme as the observed signals. As an example, *DNASE1L3* genomic region (3p14.3, rs7652027) was associated with the global disease with an OR of 1.15, whereas the OR observed for the lcSSc subtype was 1.20. Permutation analysis showed significant empirical *p* value (p* = 1.9 × 10^−3^) for *DNASE1L3*-rs7652027 thus confirming a larger effect of this risk factor in lcSSc patients (Supplementary Data 9, Supplementary Fig. 4).

Remarkably, we observed two subtype-specific signals that did not show statistical significance in the global analysis. In the case of lcSSc, there was a significant association in the *MERTK* genomic region (2q13) (association test *p* value = 1.04 × 10^−08^, OR = 1.15) that was not significant in the global meta-analysis (association test *p* value = 3.49 × 10^−05^, OR = 1.09) nor in dcSSc sub-analysis (association test *p* value = 0.503, OR = 1.02) (Supplementary Data 9 and Supplementary Fig. 5) (Supplementary Fig. 4, p* = 1.0 × 10^−4^). *MERTK* is a tyrosine kinase member of the MER/AXL/TYRO3 receptor kinase family that is associated with multiple sclerosis^27^ and hepatitis C-induced liver fibrosis^28^. Moreover, the associated variant (rs3761700), affects the expression of *MERTK* in whole blood (*p* value = 1.22 × 10^−35^).

Regarding dcSSc, we found an association signal in the *ANKRD12* genomic region (18p11.22) (association test *p* value = 3.97 × 10^−08^, OR = 1.22). This locus did not show significant associations in the global meta-analysis (association test *p* value = 2.30 × 10^−05^, OR = 1.10) nor in the lcSSc subtype (association test *p* value = 0.034, OR = 1.06) (Supplementary Data 9 and Supplementary Fig. 5) (Supplementary Fig. 4, p* = 4.0 × 10^−4^). *ANKRD12* encodes a member of the ankyrin repeats-containing cofactor family involved in the modulation of gene transcription. The associated variant—rs4798783—is an eQTL for *TWSG1* (*p* value = 6.44 × 10^−06^) in transformed fibroblasts. Interestingly, *TWSG1* enhances TGF-beta signaling (which profibrotic role is well-known) in activated T lymphocytes^29^. These findings and the specific association with dcSSc are of utmost importance, given the more aggressive and rapidly progressing fibrosis observed in this clinical subtype^2,3^.

When data were stratified by patients positive for ACA, nine genome-wide significant signals were found, of which two had been previously reported (*IRF5-TNPO3*, and *DNASE1L3*) and seven were novel associations (Supplementary Data 10). Notably, the locus *CDHR5-IRF7* was strongly associated with this autoantibody presentation (association test *p* value = 5.32 × 10^−09^, OR = 0.73) and showed stronger effect as compared to the global meta-analysis (association test *p* value = 1.97 × 10^−08^, OR = 0.80) (Supplementary Fig. 4, p* = 2.1 × 10^−3^). Regarding the ATA-positive subgroup, we replicated the previously reported association with *IRF5-TNPO3* (Supplementary Data 10)^30^. Finally, in the case of the RNA pol III-positive SSc patients, we did not observe any signal at the genome-wide significance level outside the HLA region. Nonetheless, we found suggestive associations in *FAM167A-BLK*, *GUSBP1-CDH12*, and *STEAP2* (Supplementary Data 10).

Overall, our findings highlights how performing GWASs in more homogeneous group of patients can increase the success of case–control studies by improving association strengths, thus avoiding reduction of statistical power owing to phenotypic heterogeneity^31^. Moreover, consistent with previous studies, suggesting genetic differences in the susceptibility to SSc subtypes^9,32^ the identification of specific patterns of association in each SSc subphenotype emphasizes the importance of classification biomarkers to predict more accurately the best therapeutic approach in each group of patients.

### Drug target enrichment analysis

The advantage of using human genetic evidence in drug discovery and repurposing has been comprehensively addressed in the last years^5,6^. In this line, we assessed whether any of the 78 SSc target genes identified in the present study (genes from the last three columns of Table 3) encode proteins that are drug targets in any phase of development. Seven out of the 78 genes (9%) overlapped with pharmacological active targets (*CD80*, *BLK*, *TNFSF4*, *IL12A*, *DRD4*, *PSMD3, FDFT1*) in the Open Targets Platform^33^ (Supplementary Data 11). Among them, *CD80* and *BLK* were targets of drugs for SSc in any phase of clinical trial (i.e., abatacept and dasatinib, respectively). We assessed the significance of the overlap and found that our SSc target genes were significantly enriched in pharmacological active targets for SSc (OR = 6.0; Fisher’s exact test *P* = 4.7 × 10^−2^) (Supplementary Table 5).

## Discussion

The large cohort of SSc included in the present study allowed us to identify 13 new risk loci for the disease, almost doubling the number of genome-wide association signals reported for SSc, bringing the total number of SSc risk loci up to 28.

In the present study, some of the cohorts were genotyped using different genotyping platforms between cases and controls. To control for the potential spurious associations that this fact may lead owing to the possibility of differential imputation quality for the SNPs, we applied additional steps along with the standard QC procedures. Prior to the imputation, we carefully excluded multi-allelic, or A/T-C/G variants with MAF > 0.4 from all the data sets. After imputation, we applied an in-house Perl script that compares the genotypic frequencies between cases and controls and excluded all SNPs showing genotypic inconsistencies. In addition, manual inspection of the individual Manhattan plots from the 14 independent cohorts was performed and any suspicious false positive signal was carefully analyzed and removed, if necessary. In addition, the genome-wide significant signals identified in the present study were the results of combining the effect of the signals across several independent cohorts. Moreover, no significant heterogeneity of the ORs was observed.

Applying a statistical fine-mapping approach, we reduced associated signals to 95% credible sets of 10 likely causal SNPs or fewer for 18 loci (72%). Notably, 95% credible sets comprised a single variant in 6 loci (*ARHGAP31*, *BLK*, *CD247*, *TNIP1*, *CSK*, *STAT4*-a). In other four loci, the credible sets contained two SNPs (*DGKQ*, *NUP85*-*GRB2*, *STAT4*-b, *IL12RB1*). Functional annotation of likely causal variants from credible sets revealed that all variants with PP_max_ were intronic, intergenic, or ncRNA intronic SNPs. These observations suggest that most of the genetic variations underlying SSc susceptibility are related to transcriptional regulatory mechanisms, including mRNA processing or stability mediated by ncRNAs. Our results are in accordance with emerging evidence that suggest a role of ncRNAs in autoimmunity^34^. Moreover, the exonic nonsynonymous variants included in the 95% credible sets or in high LD to credible set SNPs did not show clear evidence of being damaging mutations, as in the case of *CDHR5-*rs2740375 located close to *IRF7*. As an exception, it is worth mentioning that the nonsynonymous variant rs35677470 (R206C) in *DNASE1L3*, not present in our SNP panel, was reported to impact the DNase activity of the encoded protein in in vitro studies^35^. This fact is consistent with the result of our statistical fine-mapping since the 95% credible set for this locus was not well resolved (27 likely causal variants comprised the credible set). Further functional studies will be needed to confirm that rs35677470 (R206C) is the actual causal variant underlying the association or whether there are secondary signals that also influence the role of this genomic region in SSc susceptibility.

As expected, the results from gene expression data (eQTLs) suggested that the functional role of certain SSc signals may expand to several target genes. This hypothesis was confirmed through the experimentally derived high-resolution maps of enhancer-promoter interactions generated by H3K27ac HiChIP in human CD4^+^ T cells. On average, HiChIP results found physical interactions for approximately eight genes per SNP across the 18 SSc likely causal variants that were mapped in the H3K27ac HiChIP analysis, consistent with previous findings for other ADs^20^. Strong interactions were observed in relation to some SNPs. For example, *CD247*-rs2056626 (intronic SNP with PP_max_ = 0.99 in the fine-mapping) showed a strong normalized signal of HiChIP interaction to the *CD247* promoter, suggesting that the SNP affects an intronic regulatory element that controls gene expression. This interaction between the SSc risk SNP and the promoter of the *CD247* gene in CD4 + T-cells was also observed by the promoter capture Hi-C technique^21,36^, further supporting that the SNP may be involved in the transcriptional regulation of this gene. In fact, rs2056626 is a cis-eQTL for *CD247* (*p* value = 2.411 × 10^−48^; FDR = 0) in whole blood.

The identification of target genes for GWAS signals is one of the most challenging questions. In the present study, aggregate analysis of chromatin interaction maps in a wide spectrum of immune cell lines and eQTLs provided strong support to nominate 68 genes as robust SSc target genes (Table 3). Interestingly, the function of some of these experimentally nominated target genes are related to relevant pathways or biological processes in SSc. For example, *DDX6* encodes a RNA helicase essential for efficient miRNA-induced gene silencing. De Vries et al. demonstrated the role of DDX6 in the regulation of vascular endothelial growth factor under hypoxic conditions^37^. Therefore, this hit may establish a link between vasculopathy and SSc unknown so far.

Other two new loci providing relevant mechanistic insights are *RAB2A* and *GSDMB*. RAB2A belongs to the Rab family, a group of membrane-bound proteins, involved in vesicular fusion and trafficking. Specifically, RAB2A has been proposed to be a key factor in autophagosome clearance^38^, thus it is another SSc risk locus involved in autophagy apart from the previously described *ATG5*^9^. These results reinforce the role of autophagy in SSc pathogenesis. In regard to *GSDMB*, it encodes a member of the gasdermin-domain containing protein family. The functional mechanism of gasdermin proteins is not clearly understood yet. However, recent evidence demonstrated that some gasdermin-N domains—including *GSDMB*—play a role in the induction of pyroptosis^39,40^, an inflammatory form of cell death that is crucial for the immune response. In line of these observations, our results also suggest a role of defective pyroptosis in SSc.

Enrichment analyses of SSc loci in epigenetic marks of active gene regulation showed a strong immune signature. We identified relevant cell types and tissues for disease pathogenesis. Noteworthy, primary NK cells represented one of the highest enrichment signals across almost the entire panel. Our results are consistent with previous reports linking NK cells to SSc^41^. In a very recent publication, Benyamine et al.^42^ reported a particular expression profile of NK cells in SSc and showed that this cell type induced endothelial activation^42^. These findings may provide a link between vascular damage and the immune imbalance in SSc.

The inclusion of a wide panel of tissue and cell types captured cell type-specific patterns of enrichment. For example, there were some cell types that showed enrichment for a single histone mark. It is important to note that these results add valuable information to design future functional studies on the basis of accurately and well-chosen cell types or tissues, thus increasing the rate of success of the experiment.

Finally, it has been demonstrated that human genetic evidence positively impacts the success rate in clinical development^5^. The drug target enrichment found in the identified SSc target genes supports that our results might also be informative in drug repurposing. As an example, the present study supports the possibility to consider ustekinumab for SSc treatment, which is a drug currently approved for related diseases, such as psoriasis, active psoriatic arthritis, and Crohn’s disease.

## Methods

### Study cohorts and GWAS quality control

This study included 14 independent epidemiological cohorts comprising a total of 28,179 unrelated and genome-wide genotyped individuals (9846 SSc) patients and 18,333 healthy controls), after genotyping quality control (QC) steps. In brief, nine new SSc GWAS cohorts and five previously published SSc GWAS cohorts^7,8^ of European ancestry were included (Spain 1, Germany 1, The Netherlands 1, USA 1, France, Spain 2, Germany 2, The Netherlands 2, USA 2, Italy, UK, Sweden, Norway and Australia/UK) (Supplementary Table 1). SSc patients fulfilled the 1980 American College of Rheumatology classification criteria for this disease or the criteria proposed by LeRoy and Medsger for early-SSc^43,44^. In addition, patients were classified as having lcSSc or dcSSc, as described in LeRoy et al.^45^ Patients were also subdivided by autoantibody status according to the presence of ACA, ATA, or ARA autoantibodies. The main clinical features are shown in Supplementary Table 4. This study complied with all relevant ethical regulations. CSIC’s Ethics Committee approved the study protocol, and written informed consent was obtained in accordance with the tenets of the Declaration of Helsinki.

Genome-wide genotyping was undertaken using the arrays specified in detail in Supplementary Table 1. Stringent QC measures were applied to all GWAS data sets as follows: SNPs with call rates < 0.98; minor allele frequencies (MAFs) < 0.01; and those that deviated from Hardy-Weinberg equilibrium (HWE; p < 0.001 in both case and control subjects) were filtered out from further analysis; samples with call rates < 0.95 were removed. The presence of relatives and/or duplicates was assessed by computing identity-by-descent (IBD) estimation using PLINK^46^. An individual from each pair of relatives (Pi_Hat > 0.45) or duplicates (Pi_Hat > 0.99) was removed. Additionally, duplicate/relatedness testing was also performed between different GWAS data sets with the same country origin.

### PC analysis and identification of outliers

To identify ancestry outliers, ~100,000 quality-filtered independent SNPs were selected from each case-control GWAS cohort. PC analysis was performed using PLINK and GCTA64 and R-base software under GNU Public license v.2. The first ten PCs per individual were calculated and plotted. Samples showing > 4 standard deviations from the cluster centroids of each cohort were considered outliers and removed from further analyses.

The total number of individuals that remained in the final filtered data sets after this procedure was 26,679 (9095 SSc patients and 17,584 healthy controls).

### Imputation

QC**-**filtered GWAS data sets were subjected to whole-genome genotype imputation using IMPUTE2^47^ and the 1000 Genome Project Phase III (1KGPh3) data as reference panel^48^. GTOOL was used to convert data sets into the file format used by IMPUTE2. SNPs that were duplicated, multi-allelic, or A/T-C/G with MAF > 0.4 were excluded. Imputation was done separately for each independent study. A probability threshold of 0.9 was set for merging genotypes using GTOOL. Imputed data sets were also QC-filtered by removing SNPs with call rates < 0.98, with MAFs < 0.01 and those that deviated from HWE (*p* < 0.001). In addition, singleton SNPs (which are not informative for phasing) and those that showed genotypic inconsistency between cases and controls were also excluded from analysis using an *in house* Perl script.

### Genome-wide association analysis

Genome-wide association analyses were performed in PLINK^46^ using a logistic regression model of additive effects, including sex and the five first PCs as covariates in each of the 14 independent European cohorts. Genomic inflation factor (*λ*) was calculated by cohort and rescaled for an equivalent study of 1000 cases and 1000 controls when necessary (*λ*_1000_). Quantile–quantile (Q–Q) plots were generated and plotted with an *in house* R script to compare genome-wide distribution of the test statistic with the expected null distribution (Supplementary Fig. 1). We conducted a fixed effects inverse variance meta-analysis in PLINK^46^ to combine the ORs obtained in each independent GWAS study. Heterogeneity values (*I*^2^ and *Q*) were calculated with PLINK^46^ to evaluate possible OR heterogeneity across the 14 individuals cohorts. Novel signals of associations were defined as the genome-wide significant associations (*p* value ≤ 5 × 10^−8^) that did not overlap with previously SSc reported signals at the genome-wide significance level of association.

### Stratified analysis in clinical and serological SSc subtypes

Stratification of patients according to SSc subtype (lcSSc, dcSSc) or autoantibody status (ACA, ATA, and ARA) was performed to conduct stratified genome-wide association analyses using the same procedure as for global analysis. All sub-analyses included the 14 independent cohorts, with the exception of the GWAS analysis for ARA-positive patients, which included Spain 2, USA 2, Italy, and UK cohorts according to data availability.

### Permutation analysis for subphenotype hits

A number of loci exhibited stronger genetic signals in stratified analysis (lcSSc, dcSSc, ACA) as compared with SSc as a whole despite the loss of statistical power caused by smaller numbers of the subphenotypes. To investigate whether these outcomes could have occurred by chance, we randomly shuffled 10,000 times a number of cases from each cohort (while keeping controls constant) and reran association testing and subsequent meta-analysis on the reshuffled data sets. The *p* values were converted to *z* scores to generate a null distribution of this test statistic. In detail, for each subtype, the number of cases randomly selected was determined by the prevalence of the subtype observed in the present study: we selected 62.52% of cases for lcSSc, 27.75% for dcSSc, and 36.77% for ACA +. The empirical *p* value (p*) was calculated as the number of permuted *z* scores that were at least as extreme as the actual *z* score + 1 divided by the number of permutations + 1^31^.

### Stepwise conditional analysis in SSc-associated loci

The presence of independent signals in the genomic regions with significant signals in the meta-analysis was assessed by joint conditional analysis by GCTA^49^. This method uses summary-level statistics from meta-analysis and applies LD correction between SNPs estimated from a reference sample set. Conditional analysis of each associated locus was performed within a standard region of 1.5 Mb-window centered on the most associated SNP (index or lead SNP), with the exception of *DNASE1L3* region, where we explored the locus to 2 Mb owing to the extent of the haplotype block. LD patterns were estimated using genotype data from the 14 individual cohorts as reference. Conditional association analysis was performed including the lead SNP as covariate. Any SNP showing a conditional association *p* value < 5 × 10^−6^ was considered as independent signal and was further included in a new round of conditional analysis. This process was repeated until no SNP with *p* value < 5 × 10^−6^ remained in any of the genomic regions explored. The observed independent signals were confirmed using PLINK^46^ by dependence analysis at cohort level scans through stepwise logistic regression with adjustment for the most associated signals in each locus, followed by inverse variance weighted meta-analysis under a fixed effects model.

### Fine-mapping of SSc-associated loci in a Bayesian framework

After the assessment of independent signals in significant loci from the meta-analysis, statistical fine-mapping was carried out using PAINTOR (Probabilistic Annotation INTegratOR) v3.0^50,51^ searching for one causal SNP per independent associated region. PAINTOR performs probabilistic inference and computes posterior probabilities (PP) for SNPs to be casual considering the strength of association (*Z* score) and the LD pattern across genomic regions. The association strength was quantified using Wald statistic (“Equation (1)”) from ref. ^50^, and the LD information was provided by a LD matrix containing pairwise Pearson correlations coefficients between each SNP. In addition, PAINTOR leverages functional annotation data as a prior probability to improve SNP prioritization. Finally, the method uses Bayes theorem to obtain PP for SNPs to be casual, which in turn were used to generate 95% credible sets (the smallest list of variants that jointly have a probability of including the causal variant ≥ 95%). Associated regions that contained more than one independent signal were split to obtain regions containing only one independent signal by integrating local LD information as well as the recombination rates using the online-tool LDlink (https://ldlink.nci.nih.gov/)^52^.

The selection of functional annotations for PAINTOR fine-mapping was carried out by stratified information enrichment calculations using GARFIELD^25,26^ (http://www.ebi.ac.uk/birney-srv/GARFIELD/) (method explained in more detail in ‘Enrichment analysis of SSc risk loci in epigenetic marks and cell types’) with the annotation panel distributed in GARFIELD package. The purpose was to systematically select annotations relevant to SSc on the basis of functional enrichment analysis. GARFIELD tests its robustness by calculating functional enrichment for at least four significance cutoffs (*p* value < 1e −5/−6/−7/−8) applied to the variants. GARFIELD analysis was carried out in our genome-wide SNP panel by setting default parameters and omitting SNPs from chromosome 6 between Mb25 and Mb34. We determined a set of nine annotations to be used for fine-mapping that showed: A significant enrichment (FDR < 0.05) of GWAS SNPs for at least two out of the four significance cutoffs analyzed (*p* value < 1e −5/−6/−7/−8); and b) A low inter-annotation correlation as suggested by PAINTOR (median inter-annotation Pearson correlation  < 0.35) (Supplementary Data 12).

### Functional annotation of SNPs from credible sets

Functional characterization of the SNPs included in credible sets was performed by assessing SNP functional categories by means of wANNOVAR using default parameters^53^. Then we explored overlap with eQTLs, epigenetic histone marks of active promoters and active enhancers (H3K9ac, H3K4me1, and H3K27ac), and the presence of exonic nonsynonymous variants in high or moderate LD (*r*^2^ ≥ 0.8, *r*^2^ ≥ 0.6, respectively) using HaploReg v4.1^54^. For eQTL interrogation, we used blood eQTL from Westra et al.^55^, the Geuvadis data set^56^—which contains expression data from lymphoblastoid cell lines—and the Genotype–Tissue Expression (GTEx) project^57^—which provides RNA sequencing-based eQTL for a wide range of human tissues. Overlap of SNPs with chromatin marks was interrogated in selected cell lines from the Roadmap Epigenomics Project^15^ (Supplementary Table 1). Cell lines were selected according to the results of the functional enrichment analysis from ‘Enrichment analysis of SSc risk loci in epigenetic marks and cell types’ for H3K9ac, H3K4me1, and H3K27ac histone marks.

Finally, we assessed pleiotropic effect of our signals by determining whether the SNPs included in the credible sets had been reported to be associated with other ADs. For this, we interrogated the new NHGRI-EBI GWAS Catalog (https://www.ebi.ac.uk/gwas/)^58^ through the web tool FUMA GWAS (http://fuma.ctglab.nl/)^59^.

### H3K27ac HiChIP analysis in human CD4^+^ T cells

Experimentally derived high-resolution maps of enhancer-promoter interactions generated by H3K27ac HiChIP experiments^20^ were explored to identify target genes of SSc-associated variants. HiChIP was developed by Mumbach et al.^18^ for the analysis of protein-directed chromosome conformation in a very efficient and sensitive way. The H3K27ac HiChIP experiments were performed by Mumbach et al. in human CD4 + T cells from healthy donors: Primary human naïve T cells (CD4^+^CD45RA^+^CD25^−^CD127^hi^), regulatory T (Treg) cells (CD4^+^CD25^+^CD127^lo^) and T helper 17 (Th17) cells (CD4^+^CD45RA^−^CD25^−^CD127^hi^CCR6^+^CXCR5^−^)^20^. Virtual 4 C plots were generated from dumped matrices generated with Juicebox. The Juicebox tools dump command was used to extract the chromosome of interest from the.hic file^60,61^. The interaction profile of a specific 5 kb or 10 kb bin containing the anchor was then plotted in R. Replicate reproducibility was visualized with the mean profile shown as a line and the shading surrounding the mean representing the standard deviation between replicates. We explored chromatin interactions of the most likely causal variants by setting as anchor points the SNPs with maximum PPs in each of the independent associated loci. To identify the connectivity of candidate SNPs to target genes, we called interactions by manual inspection of individual SNP virtual 4 C interaction files and subset these interactions to those containing a transcription start site and SNP^18,20^. Fit-Hi-C algorithm was used to identify statistically significant (*q* value ≤ 1e-60) distance-matched enrichment of interaction over background^18,62^.

The functional relevance of the H3K27ac HiChIP findings was further validated by evaluating whether the explored SNPs were eQTLs for the HiChIP nominated target genes.

### Promoter capture Hi-C analysis

Chromatin interaction maps obtained by promoter capture Hi-C experiments in a wide spectrum of immune cell types^21,22^ were assessed using the web-based tool Capture Hi-C Plotter (CHiCP) (https://www.chicp.org/)^63^. The SNPs with maximum PPs in each of the independent associated loci were used as anchor points to explore physical interactions between restriction fragments containing the variants and gene promoters.

### Enrichment of SSc loci in epigenetic marks and cell types

To assess whether our SSc GWAS SNPs were not randomly distributed among functional or regulatory elements in the genome, we performed functional enrichment analysis of non-*MHC* SNPs using GARFIELD v2.0^25,26^. This method estimates enrichment of overlap on functional information computing ORs at various GWAS *p* value cutoffs, and tests the significance of the enrichment under a generalized linear model. GARFIELD accounts for major sources of confounding factors by incorporating high-LD proxies (*r*^2^ > 0.8), MAF, and transcription start site distance as categorical covariates in the logistic regression model. Enrichment was tested on independent SNPs after pruning of GWAS SNPs (*r*^2^  > 0.1). We omitted SNPs of chromosome 6 between Mb25 and Mb34 to avoid bias.

GARFIELD provides an annotation panel that includes 1005 annotations (genetic annotations, chromatin states, histone modifications, DNase I hypersensitive sites and transcription factor binding sites in different cell lines) from ENCODE, GENCODE and Roadmap Epigenomics projects^15,24,64^. Moreover, GARFIELD can be run using a custom annotation panel. The second option was selected for our enrichment analysis using annotations for 127 reference epigenomes (Supplementary Data 13) and 9 epigenetic marks (H2BK20ac, H3K27ac, H3K4me1, H3K4me2, H3K4me3, H3K9ac, H3K4ac, H3K79me1, H2BK15ac) obtained from the Roadmap Epigenomics Consortium and the Encyclopedia of DNA Elements (ENCODE) projects^15,24^. Annotations used were Imputed Narrow Peaks as generated by the software Chrom-Impute^65^ and obtained from https://egg2.wustl.edu/roadmap/data/byFileType/peaks/consolidatedImputed/narrowPeak/. The estimated ORs were computed at various GWAS *p* value cutoffs (5 × 10^−6^, 5 × 10^−7^, 5 × 10^−8^) and the R code Garfield-Meff-Padj.R provided by GARFIELD was used to calculate an enrichment *p* value threshold adjusted for multiple testing (P value = 1.25 × 10^−4^) on the effective number of annotations (Meff = 400.4454).

### Drug-target gene enrichment analysis

Target genes nominated in the present study were used to query the Open Target Platform^33^ in order to assess whether any of the genes encode proteins that are drug targets in any phase of clinical trial (phase I–IV). Enrichment of overlap between SSc target genes with pharmacological active targets for the diseases was calculated by Fisher’s exact test.

### Reporting summary

Further information on research design is available in the Nature Research Reporting Summary linked to this article.

## Supplementary information

Supplementary Information

Dataset 1

Dataset 2

Dataset 3

Dataset 4

Dataset 5

Dataset 6

Dataset 7

Dataset 8

Dataset 9

Dataset 10

Dataset 11

Dataset 12

Dataset 13

Reporting Summary

Description of Additional Supplementary Files

## Data Availability

Summary statistics of the meta-GWAS analyzed in the current study will be made available through the NHGRI-EBI GWAS Catalog (https://www.ebi.ac.uk/gwas/downloads/summary-statistics) (please use ‘Systemic Sclerosis’ and/or ‘Lopez-Isac/Martin’ as search terms). Individual-level genotype data are not publicly available owing to them containing information that could compromise research participant privacy or informed consent. All other data are contained in the article file and its supplementary information or available upon reasonable request to the corresponding authors. Epigenetic annotation panel used in this study were Imputed Narrow Peaks obtained from https://egg2.wustl.edu/roadmap/data/byFileType/peaks/consolidatedImputed/narrowPeak/.
