# Supplementary material for: GWAS for systemic sclerosis identifies multiple risk loci and highlights fibrotic and vasculopathy pathways
Source: Nat Commun. 2019 Oct 31;10:4955. doi: 10.1038/s41467-019-12760-y (PMC6823490; doi:10.1038/s41467-019-12760-y)
Supplement: Supplementary file 1 — Supplementary Information [file 41467_2019_12760_MOESM1_ESM.pdf]

SUPPLEMENTARY INFORMATION

GWAS for systemic sclerosis identifies multiple risk loci and highlights fibrotic and vasculopathy pathways

Lopez-Isac *et al*.

Table of contents

Supplementary Figures

Supplementary Figure 1. Quantile-Quantile (QQ) plots from each of the individual GWAS cohort included in the meta-analysis of systemic sclerosis in 26,679 independent individuals.

Supplementary Figure 2. Locuszoom of the stepwise conditional analysis in SSc-associated loci.

Supplementary Figure 3. Functional annotation map of 95% credible set SNPs.

Supplementary Figure 4. Permutation null distributions and empirical p-values (p) for SNPs showing more powerful genetic signals in the stratified analysis.

Supplementary Figure 5. Forest plots of systemic sclerosis subtype-specific association signals.

Supplementary Tables

Supplementary Table 1. Selected cell lines from NIH Roadmap Epigenomics Project for epigenetic annotation of SNPs from credible sets.

Supplementary Table 2. Expresion Quantitavie Trait loci (eQTLs) enrichment analysis of the SNPs from credible sets.

Supplementary Table 3. Enrichment of HiChIP target genes in systemic sclerosis eQTL genes.

Supplementary Table 4. Main clinical features of systemic sclerosis patients included in this study.

Supplementary Table 5. Drug target enrichment analysis.

SUPPLEMENTARY FIGURES

Spain

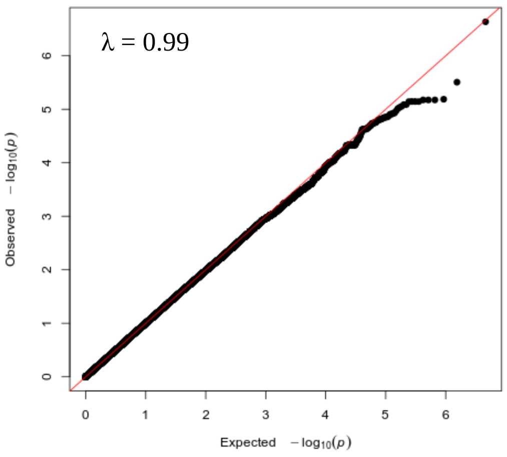

Germany 1

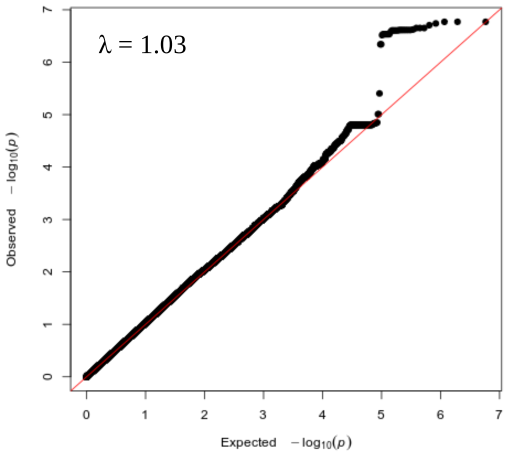

The Netherlands 1

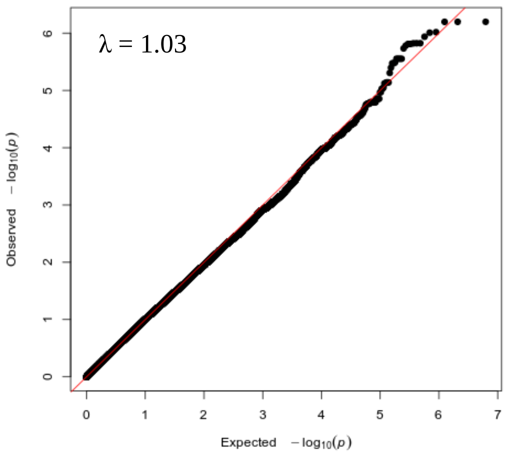

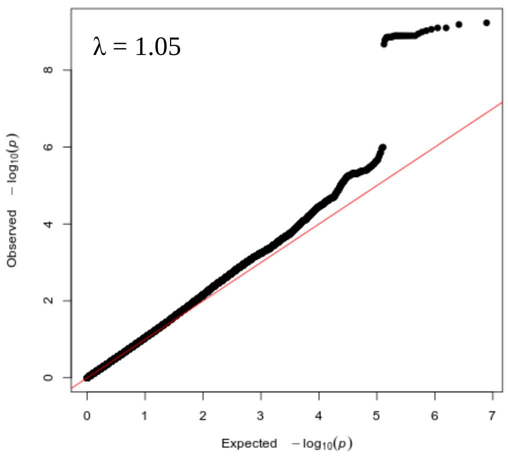

USA 1

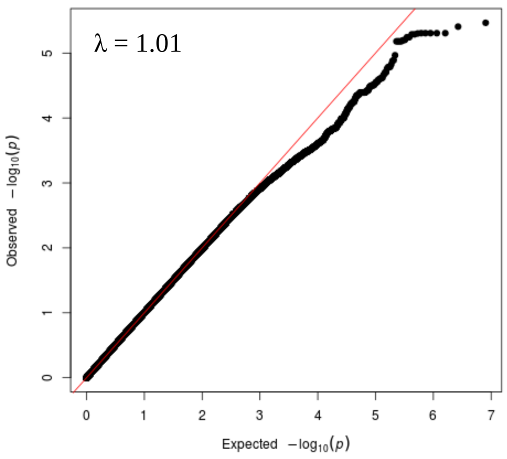

France

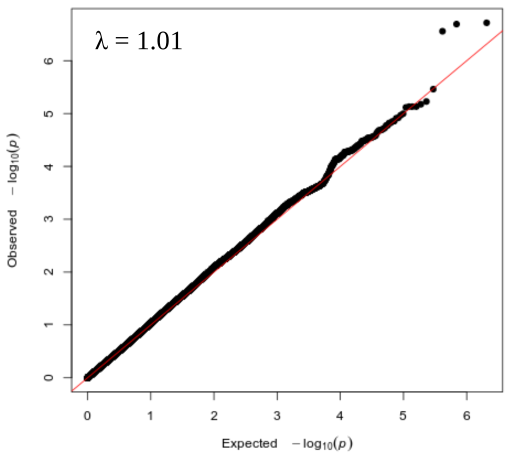

Spain 2

Germany 2 **The Netherlands 2** **USA 2**

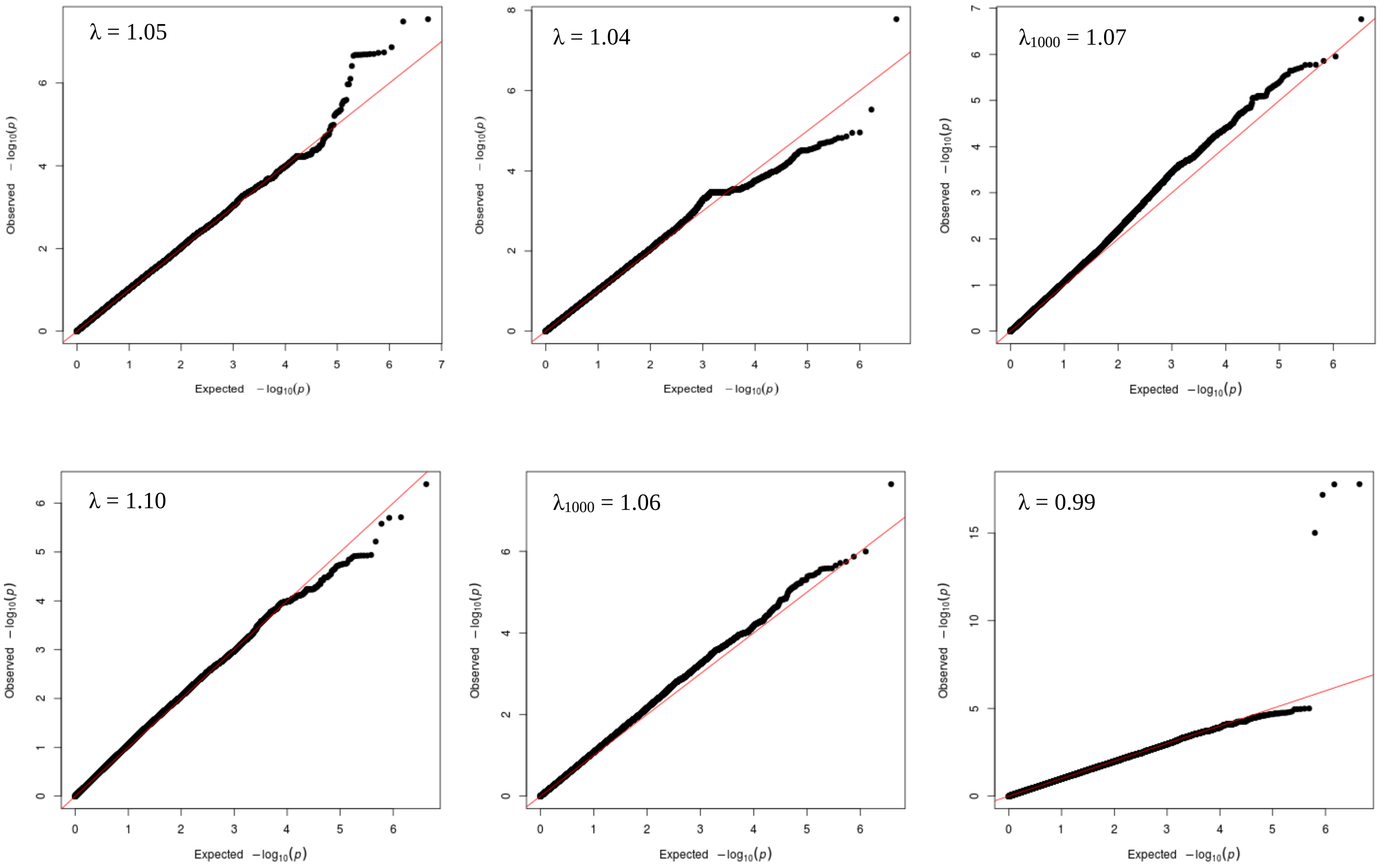

Italy **UK** **Sweden**

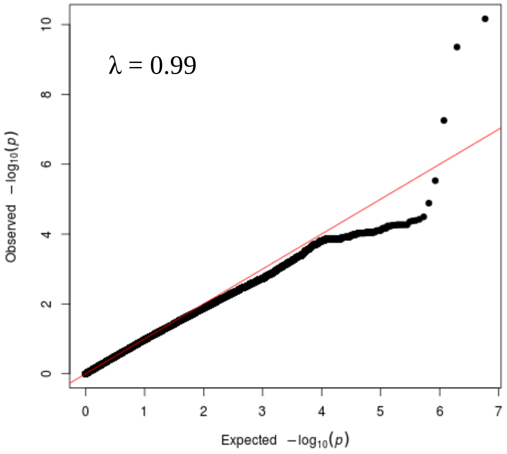

Norway

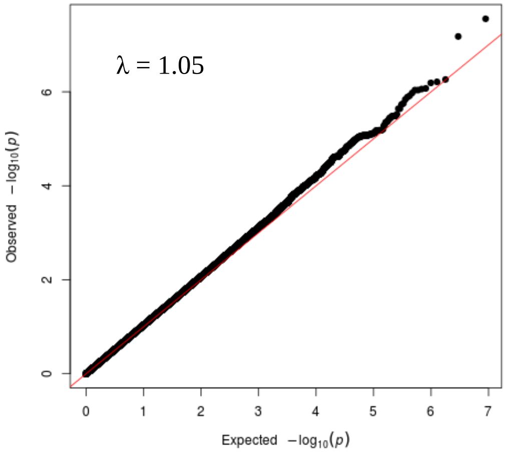

Australia

Supplementary Figure 1. **Quantile-Quantile (QQ) plots from each of the individual GWAS cohort included in the meta-analysis of systemic sclerosis in 26,679 independent individuals.** The -log10 p-values of SNP associations for each of the 14 cohorts are plotted against the expected null p-values excluding *MHC* region. The corresponding genomic inflation factors (λ) are shown in the upper part of each plot. λ calculated excluding the *MHC* region. λ_{1000} shows the λ for an equivalent study of 1,000 cases / 1,000 controls.

A)

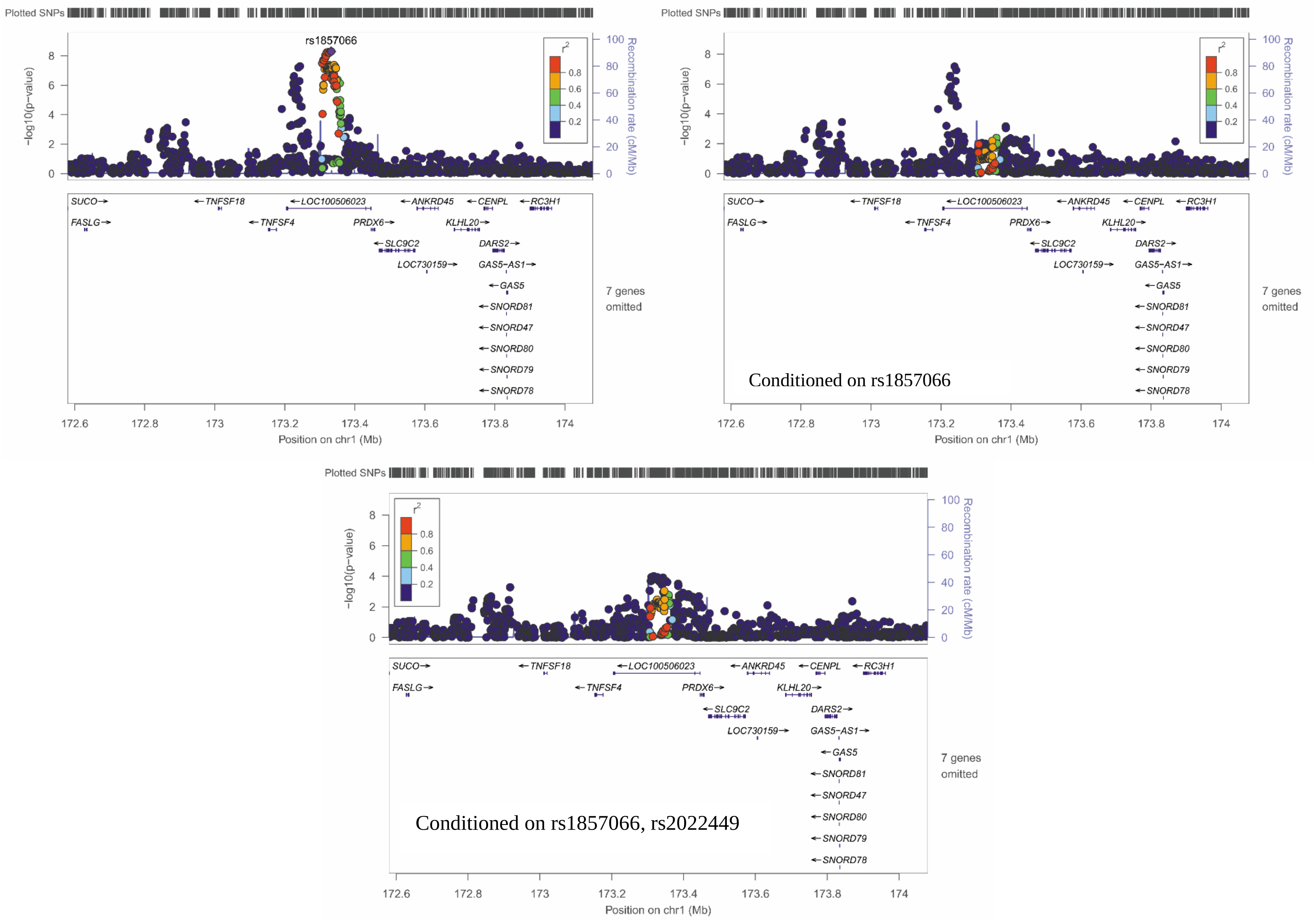
Plotted SNPs
rs1857066
−log10(p−value)
Recombination rate (cM/Mb)
r2
0.8
0.6
0.4
0.2
SUCO
FASLG
TNFSF18
TNFSF4
LOC100506023
PRDX6
SLC9C2
LOC730159
ANKRD45
KLHL20
CENPL
DARS2
GAS5−AS1
GAS5
SNORD81
SNORD47
SNORD80
SNORD79
SNORD78
RC3H1
7 genes omitted
Position on chr1 (Mb)
Conditioned on rs1857066
Conditioned on rs1857066, rs2022449

B)

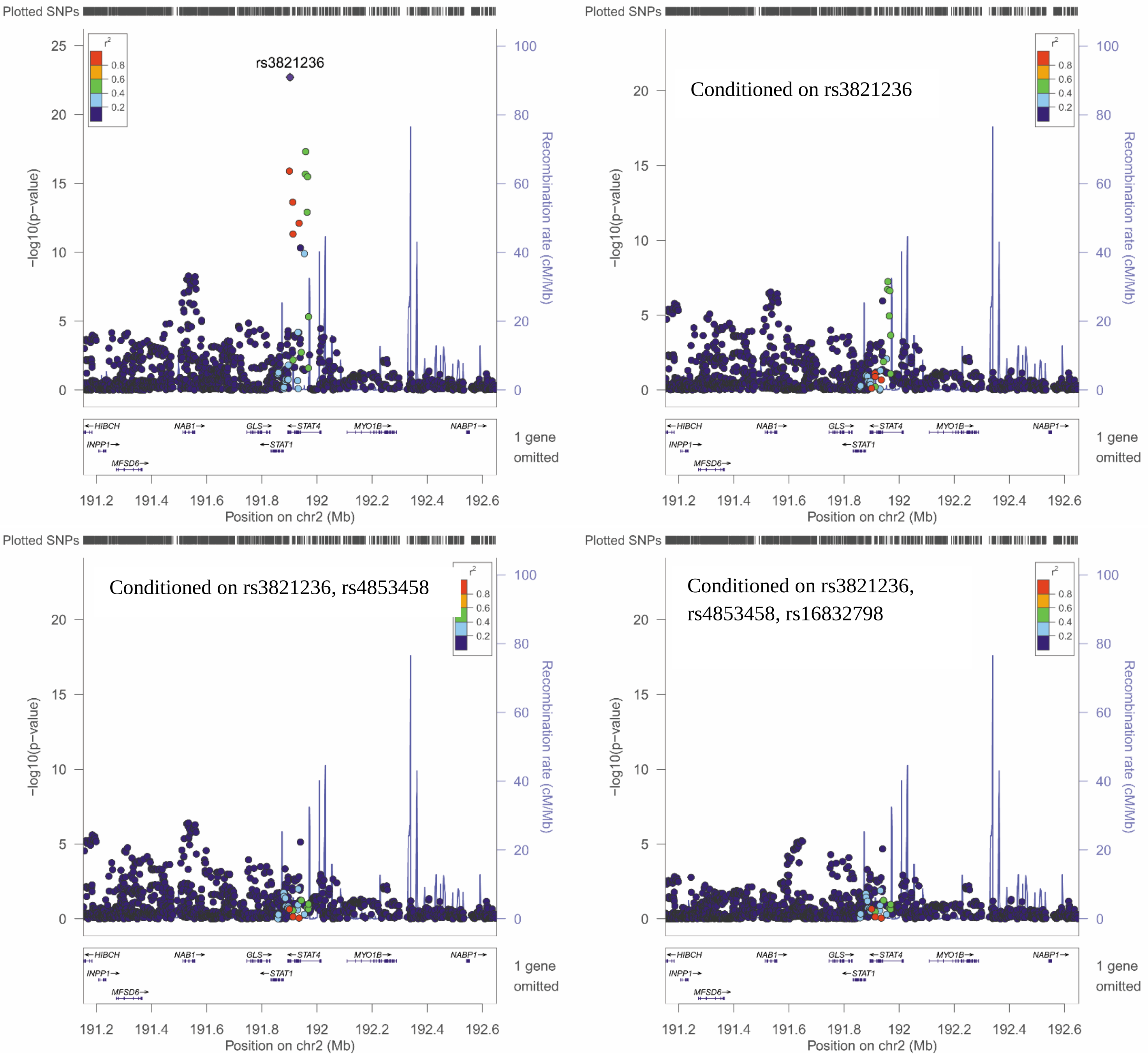
Plotted SNPs
rs3821236
r2
0.8
0.6
0.4
0.2
−log10(p−value)
Recombination rate (cM/Mb)
HIBCH
INPP1
MFSD6
NAB1
GLS
STAT4
STAT1
MYO1B
NABP1
1 gene omitted
191.2
191.4
191.6
191.8
192
192.2
192.4
192.6
Position on chr2 (Mb)
Conditioned on rs3821236
Conditioned on rs3821236, rs4853458
Conditioned on rs3821236,
rs4853458, rs16832798

C)

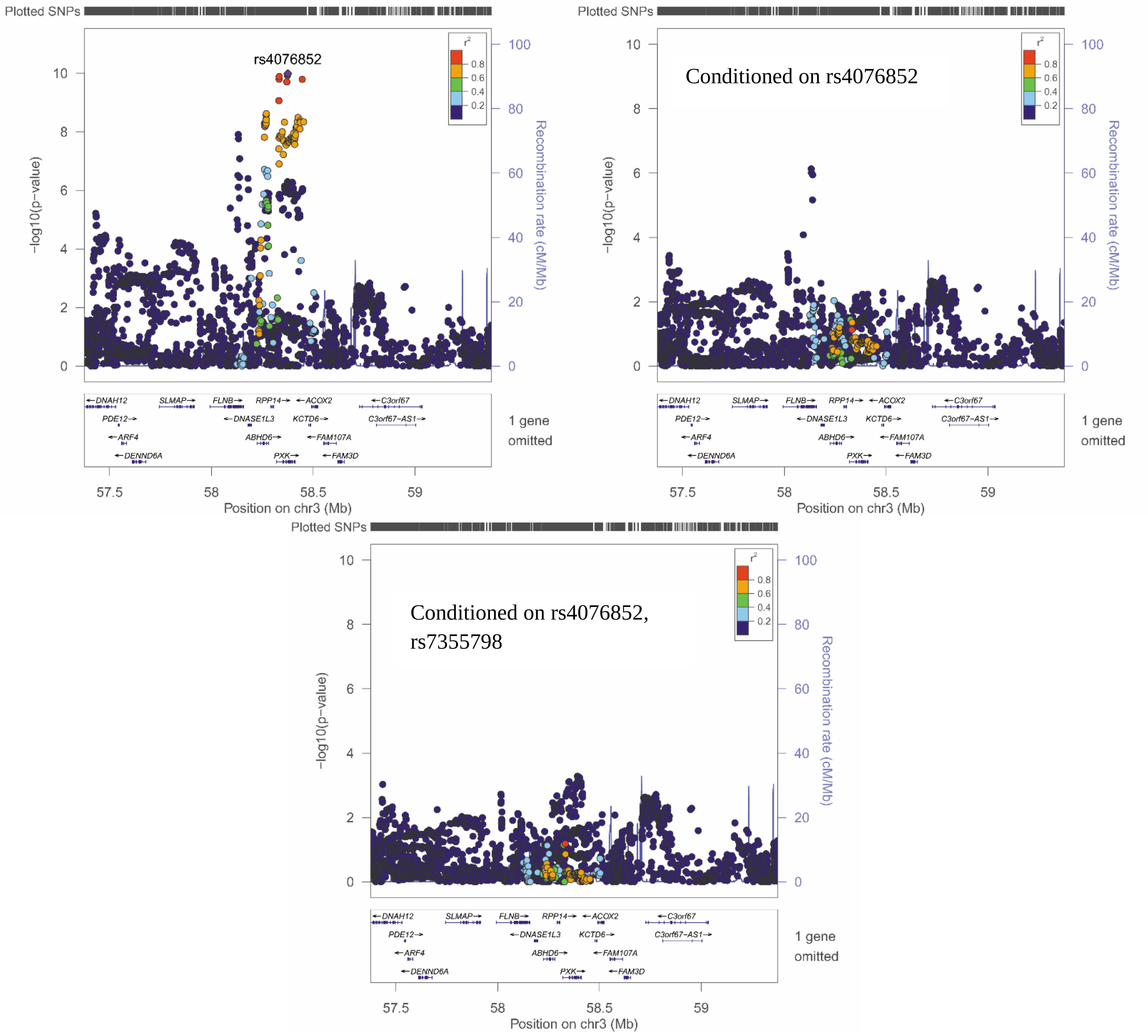

Plotted SNPs
rs4076852
r²
0.8
0.6
0.4
0.2
−log10(p−value)
Recombination rate (cM/Mb)
DNAH12
SLMAP
FLNB
RPP14
ACOX2
C3orf67
PDE12
DNASE1L3
KCTD6
C3orf67−AS1
ARF4
ABHD6
FAM107A
DENND6A
PXK
FAM3D
1 gene omitted
57.5
58
58.5
59
Position on chr3 (Mb)
Plotted SNPs
Conditioned on rs4076852
−log10(p−value)
Recombination rate (cM/Mb)
1 gene omitted
Position on chr3 (Mb)
Plotted SNPs
Conditioned on rs4076852, rs7355798
−log10(p−value)
Recombination rate (cM/Mb)
1 gene omitted
Position on chr3 (Mb)

D)

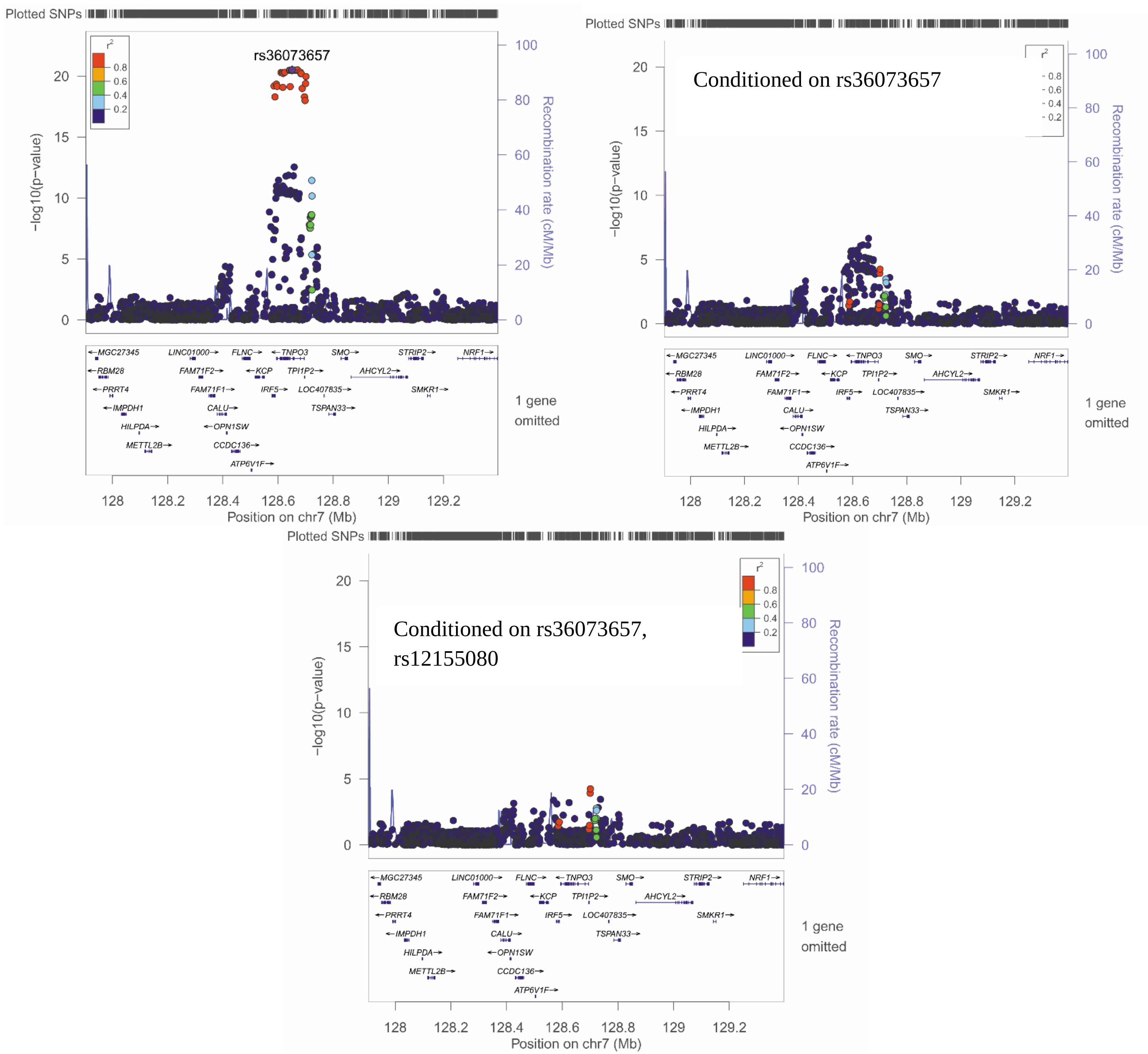

Supplementary Figure 2. Locuszoom of the stepwise conditional analysis in SSc-associated loci. A) *TNFSF4* (1q25.1) region; B) *STAT4* (2q32.2-q32.3) region; C) *DNASE1L3* (3p14.3) region; D) *IRF5-TNPO3* (7q32.1) region.

Credible-set locus	Chr	N SNPs Cred. Set	SNPs credible sets	SNP function	Gene.refGene	Exonic nonsynonymous	eQTL	H3K4me1_Enh	H3K27ac_Enh	H3K9ac_Pro	Other AID
IL12RB2	1	6	rs3790567	intronic	*IL12RB2*						
			rs3977726	intronic	*IL12RB2*						
			**rs3790566**	intronic	*IL12RB2*						
			rs10889683	intronic	*IL12RB2*						
			rs3828068	intronic	*IL12RB2*						
			rs6672670	intronic	*IL12RB2*						
CD247	1	1	**rs2056626**	intronic	*CD247*						
TNFSF4-LOC100506023-PRDX6	1	6	**rs2022449**	ncRNA_intronic	*LOC100506023*						
			rs844663	ncRNA_intronic	*LOC100506023*						
			rs12048385	ncRNA_intronic	*LOC100506023*						
			rs2840317	ncRNA_intronic	*LOC100506023*						
			rs844660	ncRNA_intronic	*LOC100506023*						
			rs844659	ncRNA_intronic	*LOC100506023*						
TNFSF4-LOC100506023-PRDX6	1	43	rs11576547	ncRNA_intronic	*LOC100506023*						
			**rs1857066**	ncRNA_intronic	*LOC100506023*						
NAB1	2	11	rs716254	intronic	*NAB1*						
			**rs16832798**	intronic	*NAB1*						
			rs4853726	intronic	*NAB1*						
			rs4853725	intronic	*NAB1*						
			rs55999263	intronic	*NAB1*						
			rs2286895	intronic	*NAB1*						
			rs3771317	intronic	*NAB1*						
			rs1860849	intronic	*NAB1*						
			rs1990462	intergenic	*NAB1;GLS*						
			rs16832836	downstream	*NAB1*						
			rs60518431	intronic	*NAB1*						
STAT4-a [1]	2	1	**rs3821236**	intronic	*STAT4*						
STAT4-b [2]	2	2	**rs4853458**	intronic	*STAT4*						
			rs7568275	intronic	*STAT4*						
FLNB-DNASE1L3-PXK	3	6	**rs7355798**	intronic	*FLNB*						
			rs9809281	intronic	*FLNB*						
			rs67418699	intronic	*FLNB*						
			rs9826147	intronic	*FLNB*						
			rs13095822	intronic	*FLNB*						
			rs9884098	intronic	*FLNB*						
FLNB-DNASE1L3-PXK	3	27	rs7653734	intronic	*PXK*						
			**rs4076852**	intronic	*PXK*						
POGLUT1-TIMMDC1-CD80-ARHGAP31	3	1	**rs9884090**	intronic	*ARHGAP31*						
IL12A	3	23	**rs589446**	ncRNA_intronic	*IL12A-AS1*						
DGKQ	4	2	**rs11724804**	intronic	*DGKQ*						
			rs13101828	intronic	*DGKQ*						
NFKB1	4	6	rs230517	intronic	*NFKB1*						
			rs230526	intronic	*NFKB1*						
			**rs230534**	intronic	*NFKB1*						
			rs230521	intronic	*NFKB1*						
			rs230528	intronic	*NFKB1*						
			rs170731	intronic	*NFKB1*						

Credible-set locus	Chr	N SNPs Cred. Set	SNPs credible sets	SNP function	Gene.refGene	Exonic nonsynonymous	eQTL	H3K4me1_Enh	H3K27ac_Enh	H3K9ac_Pro	Other AID
TNIP1	5	1	**rs3792783**	intronic	*TNIP1*						
ATG5	6	3	**rs633724**	intronic	*ATG5*						
			rs9486314	intronic	*ATG5*						
			rs11752888	intronic	*ATG5*						
IRF5-TNPO3	7	NA	**rs36073657**	intronic	*TNPO3*						
		NA	**rs12155080**	intronic	*TNPO3*						
FAM167A-BLK	8	1	**rs2736340**	intergenic	*FAM167A;BLK*						
RAB2A-CHD7	8	80	rs6987084	intronic	*RAB2A*						
			**rs685985**	intergenic	*RAB2A;CHD7*						
CDHR5-IRF7	11	4	**rs6598008**	intronic	*CDHR5*						
			rs2740380	exonic	*CDHR5*						
			rs2740375	exonic	*CDHR5*						
			rs702966	UTR3	*PHRF1*						
TSPAN32,CD81-AS1	11	20	**rs2651804**	intergenic	*TSPAN32;CD81-AS1*						
DDX6	11	7	rs10892286	intronic	*DDX6*						
			rs10892288	intronic	*DDX6*						
			**rs11217020**	intronic	*DDX6*						
			rs874621	intronic	*DDX6*						
			rs10892292	intronic	*DDX6*						
			rs11826521	intergenic	*TREH;DDX6*						
			rs10892280	intergenic	*TREH;DDX6*						
CSK	15	1	**rs1378942**	intronic	*CSK*						
IRF8	16	6	rs11117422	intergenic	*IRF8;LINC01082*						
			**rs11117420**	intergenic	*IRF8;LINC01082*						
			rs13335265	intergenic	*IRF8;LINC01082*						
			rs11644034	intergenic	*IRF8;LINC01082*						
			rs12711490	intergenic	*IRF8;LINC01082*						
			rs4843323	intergenic	*IRF8;LINC01082*						
IKZF3-GSDMB	17	17	rs9303277	intronic	*IKZF3*						
			**rs883770**	intronic	*GSDMB*						
NUP85-GRB2	17	2	**rs1005714**	intronic	*NUP85*						
			rs9909306	intronic	*NUP85*						
IL12RB1	19	2	**rs2305743**	intronic	*IL12RB1*						
			rs12150884	intronic	*IL12RB1*						

Supplementary Figure 3. Functional annotation map of 95% credible set SNPs. In each category, dark colors represent overlap with the SNP, and light colors indicate overlap with proxy SNPs. For 'exonic non-synonymous' category, medium-light and lightest colors indicate SNPs in high to moderate LD ($r2 \geq 0.8$, $r2 \geq 0.6$, respectively). Supplementary table 3 provides the cell types that were used to identify overlap with chromatin marks of active enhancers (H3K4me1, H3K27ac) and active promoters (H3K9ac). When the 95% credible set was not well resolved (credible sets that contained more than 15 likely causal variants), we selected the SNP with PP_{max} and the index SNP. In the case of *IRF5-TNPO3*, where the credible set was not feasible, we selected the two independent signals identified at this locus. Index SNPs are highlighted in bold.

A)

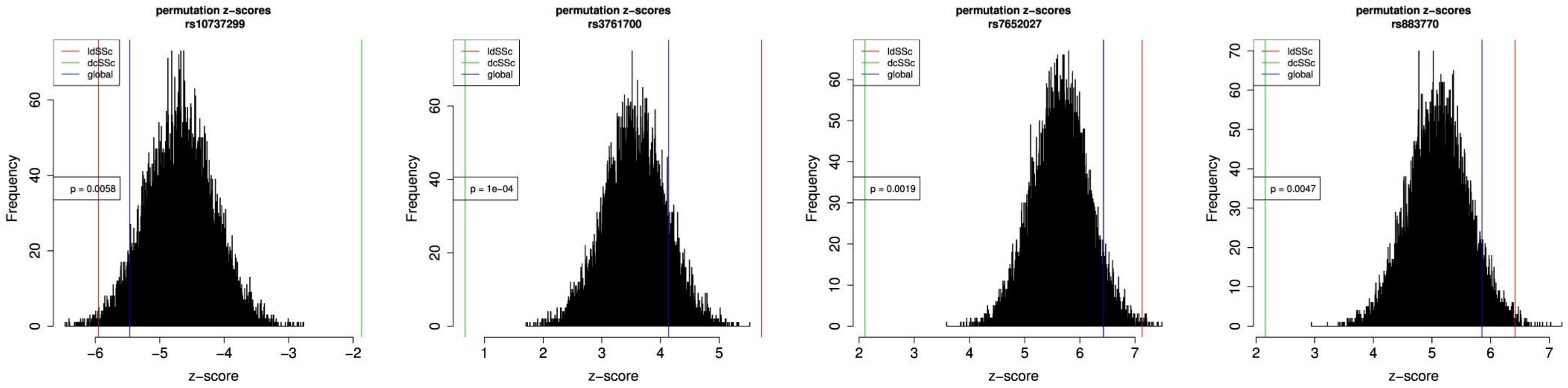

B)

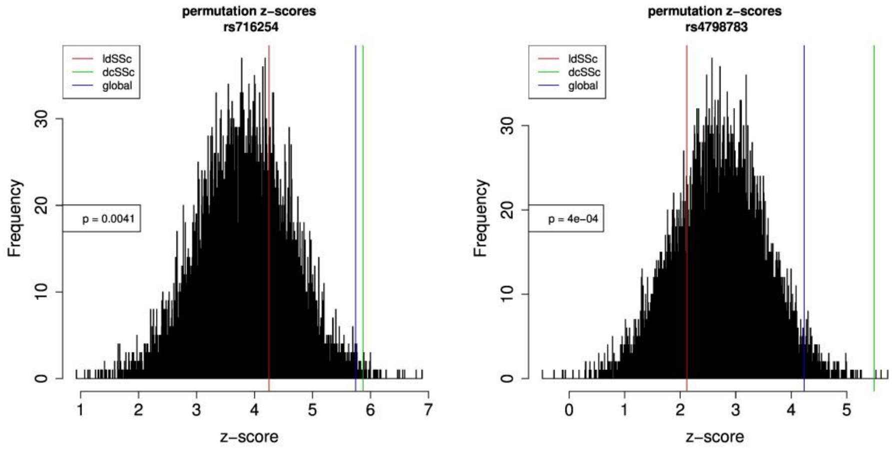

C)

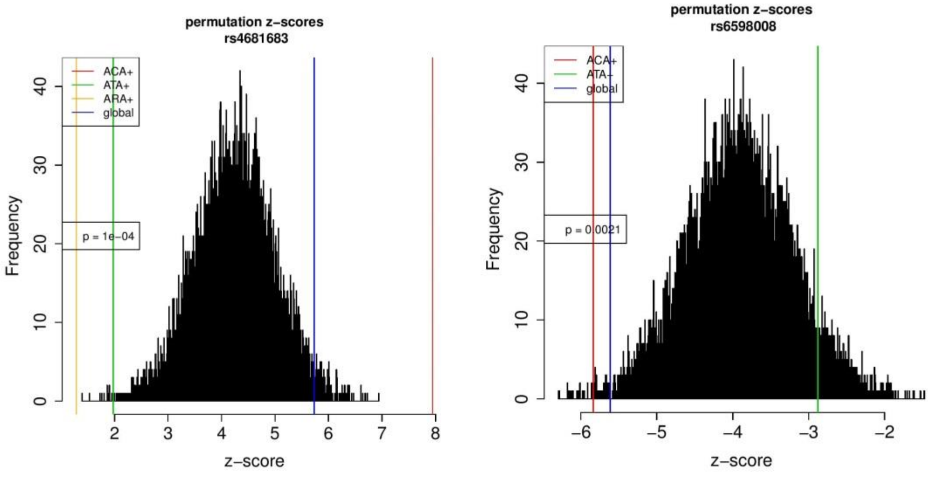

Supplementary Figure 4. Permutation null distributions and empirical p-values (p) for SNPs showing more powerful genetic signals in the stratified analysis. A) Limited cutaneous SSc (lcSSc); B) Diffuse cutaneous SSc (dcSSc); C) Anticentromere autoantibodies (ACA) subgroup.

A)

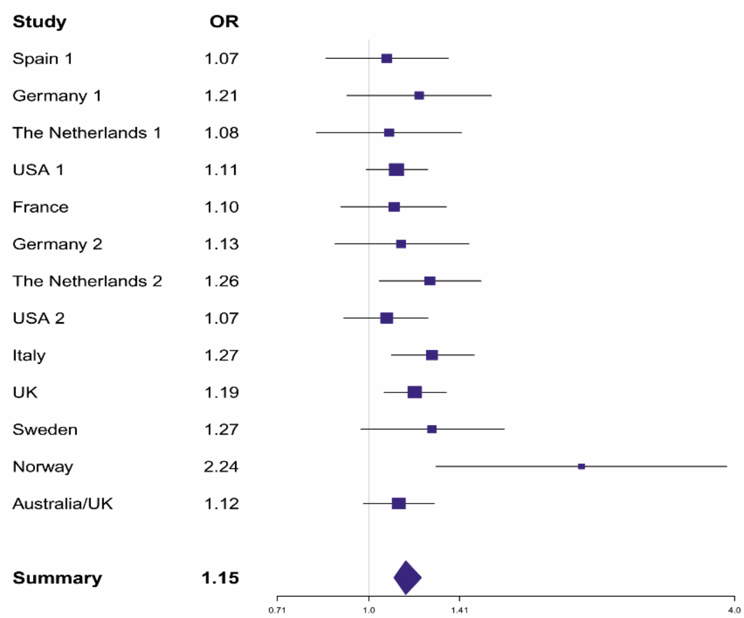

B)

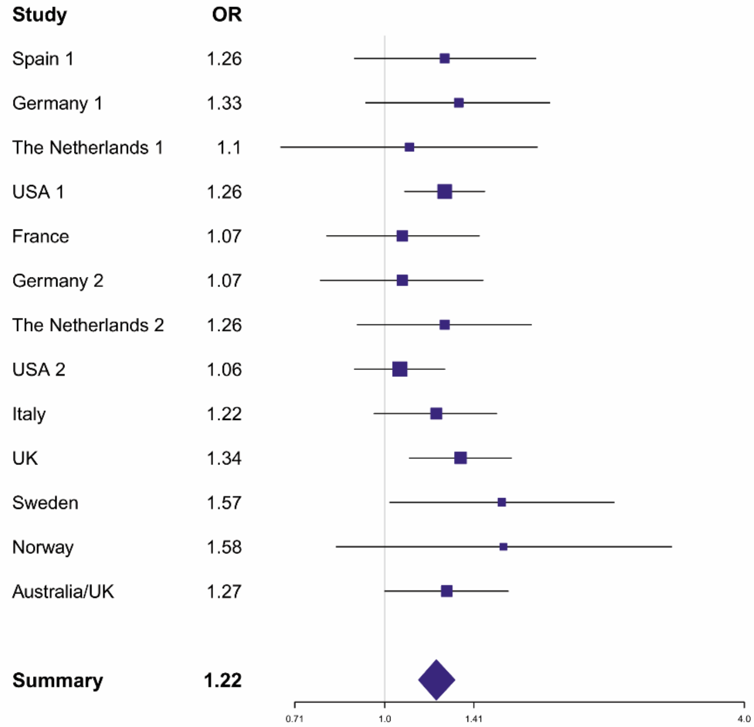

Supplementary Figure 5. Forest plots of systemic sclerosis subtype-specific association signals. *MERTK*-rs3761700 (A) and *ANKRD12* -rs4798783 (B) association signals from the stratified analyses by clinical subtypes (lcSSc and dcSSc, respectively).

Supplementary Tables

Supplementary Table 1. Selected cell lines from NIH Roadmap Epigenomics Project for epigenetic annotation of SNPs from credible sets.

Epigenome ID (EID)	Abreviation	Description
E006	ESDR.H1.MSC	H1 Derived Mesenchymal Stem Cells
E017	LNG.IMR90	IMR90 fetal lung fibroblasts Cell Line
E029	BLD.CD14.PC	Primary monocytes from peripheral blood
E030	BLD.CD15.PC	Primary neutrophils from peripheral blood
E031	BLD.CD19.CPC	Primary B cells from cord blood
E032	BLD.CD19.PPC	Primary B cells from peripheral blood
E033	BLD.CD3.CPC	Primary T cells from cord blood
E034	BLD.CD3.PPC	Primary T cells from peripheral blood
E035	BLD.CD34.PC	Primary hematopoietic stem cells
E036	BLD.CD34.CC	Primary hematopoietic stem cells short term culture
E037	BLD.CD4.MPC	Primary T helper memory cells from peripheral blood 2
E038	BLD.CD4.NPC	Primary T helper naive cells from peripheral blood
E039	BLD.CD4.CD25M.CD45RA.NPC	Primary T helper naive cells from peripheral blood
E040	BLD.CD4.CD25M.CD45RO.MPC	Primary T helper memory cells from peripheral blood 1
E041	BLD.CD4.CD25M.IL17M.PL.TPC	Primary T helper cells PMA-I stimulated
E042	BLD.CD4.CD25M.IL17P.PL.TPC	Primary T helper 17 cells PMA-I stimulated
E043	BLD.CD4.CD25M.TPC	Primary T helper cells from peripheral blood
E044	BLD.CD4.CD25.CD127M.TREGPC	Primary T regulatory cells from peripheral blood
E045	BLD.CD4.CD25I.CD127.TMEMPC	Primary T cells effector/memory enriched from peripheral blood
E046	BLD.CD56.PC	Primary Natural Killer cells from peripheral blood
E047	BLD.CD8.NPC	Primary T CD8+ naive cells from peripheral blood
E048	BLD.CD8.MPC	Primary T CD8+ memory cells from peripheral blood
E050	BLD.MOB.CD34.PC.F	Primary hematopoietic stem cells G-CSF-mobilized Female
E051	BLD.MOB.CD34.PC.M	Primary hematopoietic stem cells G-CSF-mobilized Male
E055	SKIN.PEN.FRSK.FIB.01	Foreskin Fibroblast Primary Cells skin01
E056	SKIN.PEN.FRSK.FIB.02	Foreskin Fibroblast Primary Cells skin02
E062	BLD.PER.MONUC.PC	Primary mononuclear cells from peripheral blood
E093	THYM.FET	Fetal Thymus
E112	THYM	Thymus
E113	SPLN	Spleen
E114	LNG.A549.ETOH002.CNCR	A549 EtOH 0.02pct Lung Carcinoma Cell Line
E115	BLD.DND41.CNCR	Dnd41 TCell Leukemia Cell Line
E116	BLD.GM12878	GM12878 Lymphoblastoid Cells
E123	BLD.K562.CNCR	K562 Leukemia Cells
E124	BLD.CD14.MONO	Monocytes-CD14+ RO01746 Primary Cells
E129	BONE.OSTEO	Osteoblast Primary Cells

Supplementary Table 2. Expresion Quantitavie Trait loci (eQTLs) enrichment analysis of the SNPs from credible sets.

Number of SSc 'credible set SNPs' that are blood eQTLs	61
Number of SSc 'credible set SNPs' that are not blood eQTLs	20
Total number of blood eQTLs[a]	1,181,655
**Fisher exact test P-value[c]**	**5.65E-06**
Number of SSc 'credible set SNPs' that are non-blood eQTLs	50
Number of SSc 'credible set SNPs' that are not non-blood eQTLs	31
Total number of non-blood eQTLs[b]	1,293,910
**Fisher exact test P-value[c]**	**4.48E-02**

[a]eQTLs obtained from Westra et al. (PMID: 24013639), the Geuvadis dataset (PMID: 24037378), and the Genotype–Tissue Expression (GTEx) project (PMID: 25954001) (only blood).

[b]eQTLs obtained from GTEx (PMID: 25954001) considering the following tissues: Artery, Fibroblasts, Colon, Intestine, Esophagus, Lung, Skeletal Muscle, Skin.

[c]Fisher exact tests were calculated assuming 50% of the project-assayed SNPs being significant eQTLs (GTEx Consortium, PMID: 29022597).

Supplementary Table 3. Enrichment of HiChIP target genes in systemic sclerosis eQTL genes.

Number of SSc eQTL genes overlapping nominated HiChIP target genes	40
Number of SSc eQTL genes not overlapping nominated HiChIP target genes	42
Number of nominated HiChIP target genes that are not SSc eQTL genes	114
Number of genes within ±1 Mb window centered on 27 SSc SNPs[1,2]	1,209
**Fisher exact test P-value**	**2.92E-19**

[1]Number of genes within ±1 Mb window centered on 27 SSc SNPs that were not SSc eQTL genes nor nominated HiChIP target genes.

[2]Single-nucleotide polymorphisms (SNPs) with the maximum posterior probability (PPmax) from the 27 loci independently associated to systemic sclerosis (SSc).

Supplementary Table 4. Main clinical features of systemic sclerosis patients included in this study.

GWAS cohort	lcSSc (%)	dcSSc (%)	With lcSSc/dcSSc (%)	ACA+ (%)	With ACA data (%)	ATA+ (%)	With ATA data (%)	ARA+ (%)	With ARA data (%)	Total Cases
**Spain 1**	220 (60.94)	90 (24.93)	310 (85)	170 (47.09)	329 (91)	80 (22.16)	320 (88)	NA	NA	361
**Germany 1**	148 (57.58)	100 (38.91)	248 (96)	116 (45.13	249 (96)	76 (29.57)	245 (95)	NA	NA	257
**Netherlands 1**	125 (68.30)	40 (21.85)	165 (90)	42 (22.95)	166 (90)	42 (22.95)	166 (90)	NA	NA	183
**USA 1**	822 (60.21)	466 (34.13)	1288 (94)	395 (28.93)	1248 (91)	210 (15.38)	1267 (93)	NA	NA	1365
**France**	341 (63.03)	177 (32.71)	518 (95)	191 (35.30)	489 (90)	123 (22.73)	488 (90)	NA	NA	541
**Spain 2**	684 (58.51)	282 (24.12)	966 (82)	470 (40.20)	1004 (85)	221 (18.90)	990 (84)	25 (2.13)	215 (18.39%)	1169
**Germany 2**	180 (49.45)	120 (32.96)	300 (82)	133 (36.53)	331 (90)	95 (26.09)	330 (90)	NA	NA	364
**Netherlands 2**	296 (65.92)	95 (21.15)	391 (87)	143 (31.84)	380 (84)	74 (16.48)	381 (84)	NA	NA	449
**USA 2**	750 (58.32)	471 (36.62)	1221 (95)	411 (31.95)	1276 (99)	193 (15.00)	1273 (98)	218 (16.95)	1240 (96.42%)	1286
**Italy**	588 (58.91)	193 (19.33)	781 (78)	436 (43.68)	946 (95)	328 (32.86)	947 (95)	197 (19.73)	537 (53.81%)	998
**UK**	774 (70.74)	236 (21.57)	875 (80)	396 (36.19)	779 (71)	173 (15.81)	775 (70)	118 (10.78)	138 (12.61%)	1094
**Sweden**	120 (70.58)	50 (29.41)	170 (100)	44 (25.88)	168 (98)	25 (14.70)	168 (98)	NA	NA	170
**Norway**	59 (61.45)	31 (32.29)	90 (93)	49 (51.04)	88 (92)	15 (15.62)	89 (92)	NA	NA	96
**Australia/UK**	579 (75.98)	173 (22.70)	752 (98)	348 (45.66)	669 (87)	94 (12.33)	645 (84)	NA	NA	762
**Total**	5,686 (62.52)	2,524 (27.75)		3,344 (36.77)		1,749 (19.20)		558 (6.14)		9,095

Supplementary Table 5. Drug target enrichment analysis.

	**Systemic Sclerosis**
Number of related gene-products[a]	78
Number of related gene-products & drug targets[b]	2
Number of unrelated gene-products & drug targets[c]	96
Number of unrelated gene-products & no drug targets[d]	21,838
**Exact Fisher's test *p*-value**	**0.047**

[a]Number of nominated gene-products as related with systemic sclerosis.

[b]Number of nominated gene-products that are drug targets for systemic sclerosis.

[c]Gene-products that are drug target for the disease but that are not related in our study samples.

[d]Gene-products that are not related in our study neither drug target for systemic sclerosis.
