# Supplementary material for: GWAS for systemic sclerosis identifies multiple risk loci and highlights fibrotic and vasculopathy pathways
Source: Nat Commun. 2019 Oct 31;10:4955. doi: 10.1038/s41467-019-12760-y (PMC6823490; doi:10.1038/s41467-019-12760-y)
Supplement: Supplementary file 15 — Reporting Summary [file 41467_2019_12760_MOESM15_ESM.pdf]

nature research

Corresponding author(s): Elena Lopez-Isac & Javier Martin

Last updated by author(s): Sep 6, 2019

Reporting Summary

Nature Research wishes to improve the reproducibility of the work that we publish. This form provides structure for consistency and transparency in reporting. For further information on Nature Research policies, see Authors & Referees and the Editorial Policy Checklist.

Statistics

For all statistical analyses, confirm that the following items are present in the figure legend, table legend, main text, or Methods section.

n/a	Confirmed	
☐	☒	The exact sample size (*n*) for each experimental group/condition, given as a discrete number and unit of measurement
☒	☐	A statement on whether measurements were taken from distinct samples or whether the same sample was measured repeatedly
☐	☒	The statistical test(s) used AND whether they are one- or two-sided<br>*Only common tests should be described solely by name; describe more complex techniques in the Methods section.*
☐	☒	A description of all covariates tested
☐	☒	A description of any assumptions or corrections, such as tests of normality and adjustment for multiple comparisons
☐	☒	A full description of the statistical parameters including central tendency (e.g. means) or other basic estimates (e.g. regression coefficient) AND variation (e.g. standard deviation) or associated estimates of uncertainty (e.g. confidence intervals)
☐	☒	For null hypothesis testing, the test statistic (e.g. *F*, *t*, *r*) with confidence intervals, effect sizes, degrees of freedom and *P* value noted<br>*Give P values as exact values whenever suitable.*
☐	☒	For Bayesian analysis, information on the choice of priors and Markov chain Monte Carlo settings
☒	☐	For hierarchical and complex designs, identification of the appropriate level for tests and full reporting of outcomes
☐	☒	Estimates of effect sizes (e.g. Cohen's *d*, Pearson's *r*), indicating how they were calculated

Our web collection on statistics for biologists contains articles on many of the points above.

Software and code

Policy information about availability of computer code

Data collection	No software was used for data collection
Data analysis	The software used for analyses are described and/or cited in the methods (PLINK v.1.9, PLINK v.1.07, R-base software, GCTA64, IMPUTE2, GTOOL, GCTA-COJO, PAINTOR v3.0, GARFIELD, wANNOVAR, HaploReg v.4.1, blood eQTL, Genotype-Tissue Expression project (GTEx), FUMA GWAS, LD link, Juicebox, Fit-Hi-C, DEPICT, CHiCPlotter)

For manuscripts utilizing custom algorithms or software that are central to the research but not yet described in published literature, software must be made available to editors/reviewers. We strongly encourage code deposition in a community repository (e.g. GitHub). See the Nature Research guidelines for submitting code & software for further information.

Data

Policy information about availability of data

All manuscripts must include a data availability statement. This statement should provide the following information, where applicable:
- Accession codes, unique identifiers, or web links for publicly available datasets
- A list of figures that have associated raw data
- A description of any restrictions on data availability

Summary statistics of the meta-GWAS analyzed in the current study will be made available through the NHGRI-EBI GWAS Catalog (https://www.ebi.ac.uk/gwas/downloads/summary-statistics) (please use 'Systemic Sclerosis' or 'Lopez-Isac/Martin' as search terms). Individual-level genotype data are not publicly available due to them containing information that could compromise research participant privacy or informed consent. All other data are contained in the article file and its supplementary information or available upon reasonable request to the corresponding authors. Epigenetic annotation panel used in this study were Imputed Narrow Peaks obtained from https://egg2.wustl.edu/roadmap/data/byFileType/peaks/consolidatedImputed/narrowPeak/.

Field-specific reporting

Please select the one below that is the best fit for your research. If you are not sure, read the appropriate sections before making your selection.

☒ Life sciences ☐ Behavioural & social sciences ☐ Ecological, evolutionary & environmental sciences

For a reference copy of the document with all sections, see nature.com/documents/nr-reporting-summary-flat.pdf

Life sciences study design

All studies must disclose on these points even when the disclosure is negative.

Sample size	Sample size of the meta-GWAS was determined according to the availability of genome-wide genotyping data for systemic sclerosis patients and healthy controls. The study reached a total of 28,179 unrelated individuals (9,846 systemic sclerosis patients and 18,333 healthy controls), thus providing enough power to discover new loci, based on results of similar studies in similar diseases. Overall, we have 99% statistical power to detect variants with 5.5% of minor allele frequency in an additive model and a significance threshold of p < 5x10-8.
Data exclusions	Standard GWAS quality control procedures were applied for exclusion criteria. Methods describe the criteria in details.
Replication	This study includes 14 independent epidemiological cohorts and represents the largest cohort of systemic sclerosis patients in the world. Thus no reasonable replication could be performed.
Randomization	Not relevant to our study
Blinding	Not relevant to our study

Reporting for specific materials, systems and methods

We require information from authors about some types of materials, experimental systems and methods used in many studies. Here, indicate whether each material, system or method listed is relevant to your study. If you are not sure if a list item applies to your research, read the appropriate section before selecting a response.

Materials & experimental systems

n/a	Involved in the study	
☒	☐	Antibodies
☒	☐	Eukaryotic cell lines
☒	☐	Palaeontology
☒	☐	Animals and other organisms
☐	☒	Human research participants
☒	☐	Clinical data

Methods

n/a	Involved in the study	
☒	☐	ChIP-seq
☒	☐	Flow cytometry
☒	☐	MRI-based neuroimaging

Human research participants

Policy information about studies involving human research participants

Population characteristics	The 14 independent epidemiological cohorts included in this study are of European ancestry. Supplementary table 1 describes the epidemiological characteristics of each case-control collection. The main clinical features are shown in Supplementary Table 12. All the independent cohorts were subjected to Principal Component Analysis to identify population outliers and correct for potential population stratification.
Recruitment	SSc patients fulfilled the 1980 American College of Rheumatology classification criteria for this disease or the criteria proposed by LeRoy and Medsger (Anon, M.C., Arthritis Rheum, 1980; LeRoy, E.C. & Medsger, T.A., J Rheumatol, 2001) for early-SSc. In addition, patients were classified as having limited cutaneous od diffuse cutaneous SSc, as described in LeRoy et al (LeRoy, E.C. et al, J Rheumatol, 1988).
Ethics oversight	CSIC's Ethics Committee approved the study protocol, and written informed consent was obtained in accordance with the tenets of the Declaration of Helsinki.

Note that full information on the approval of the study protocol must also be provided in the manuscript.
