# Supplementary material for: GWAS for systemic sclerosis identifies multiple risk loci and highlights fibrotic and vasculopathy pathways
Source: Nat Commun. 2019 Oct 31;10:4955. doi: 10.1038/s41467-019-12760-y (PMC6823490; doi:10.1038/s41467-019-12760-y)
Supplement: Supplementary file 16 — Description of Additional Supplementary Files [file 41467_2019_12760_MOESM16_ESM.pdf]

Supplementary Data Information

Title: Supplementary Data 1.
Description: Summary of the study cohorts.

Title: Supplementary Data 2.
Description: Credible sets of the 25 fine-mapped loci.

Title: Supplementary Data 3.
Description: Expresion Quantitavie Trait loci (eQTLs) of the SNPs from credible sets.

Title: Supplementary Data 4.
Description: Exonic non-synonymous variants from credible sets or exonic non-synonymous variants in high to moderate LD ($r2 \geq 0.8$, $r2 \geq 0.6$, respectively) with SNPs from credible sets.

Title: Supplementary Data 5.
Description: SNPs from credible sets showing pleiotropic effect in other diseases.

Title: Supplementary Data 6.
Description: Promoter capture Hi-C target genes in immune cell types and eQTL validation for the 27 systemic sclerosis association signals.

Title: Supplementary Data 7.
Description: Gene prioritization by means of DEPICT.

Title: Supplementary Data 8.
Description: Significant enrichments for epigenetic marks in 59 cell/tissue types analyzed.

Title: Supplementary Data 9.
Description: Non-HLA loci Associated with SSc clinical subphenotypes.

Title: Supplementary Data 10.
Description: Non-HLA loci associated with SSc autoantibody status.

Title: Supplementary Data 11.
Description: Gene-products nominated in the meta-analysis for systemic sclerosis and their related drugs.

Supplementary Data 12. Summary of the functional annotation data integrated in the statistical fine-mapping.

Supplementary Data 13. Reference epigenomes from the Roadmap Epigenomics Consortium and the Encyclopedia of DNA Elements (ENCODE) projects.
